# Association between genetic polymorphisms in cytochrome P450 enzymes and survivals in women with breast cancer receiving adjuvant endocrine therapy: a systematic review and meta-analysis

**DOI:** 10.1017/erm.2021.28

**Published:** 2022-01-07

**Authors:** Carmen Wing Han Chan, Caixia Li, Eleven Jinnan Xiao, Minjie Li, Patrick Gladson McLeywick Phiri, Tingting Yan, Judy Yuet Wa Chan

**Affiliations:** 1 The Nethersole School of Nursing, Faculty of Medicine, The Chinese University of Hong Kong, Hong Kong SAR, China; 2 Xiangya School of Nursing, Central South University, Changsha, China; 3 Nursing Department, Shaanxi Provincial People's Hospital, Xi'an Shaanxi, China

**Keywords:** Adjuvant endocrine therapy, breast cancer, cytochrome P450, genetic polymorphism, genotype, survival, tamoxifen

## Abstract

Tamoxifen is commonly prescribed for preventing recurrence in patients with breast cancer. However, the responses of the patients on tamoxifen treatment are variable. Cytochrome P450 genetic variants have been reported to have a significant impact on the clinical outcomes of tamoxifen treatment but no tangible conclusion can be made up till now. The present review attempts to provide a comprehensive review on the associative relationship between genetic polymorphisms in cytochrome P450 enzymes and survival in breast cancer patients on adjuvant tamoxifen therapy. The literature search was conducted using five databases, resulting in the inclusion of 58 studies in the review. An appraisal of the reporting quality of the included studies was conducted using the assessment tool from the Effective Public Health Practice Project (EPHPP). Meta-analyses were performed on CYP2D6 studies using Review Manager 5.3 software. For other studies, descriptive analyses were performed. The results of meta-analyses demonstrated that shorter overall survival, disease-free survival and relapse-free survival were found in the patients with decreased metabolisers when compared to normal metabolisers. The findings also showed that varying and conflicting results were reported by the included studies. The possible explanations for the variable results are discussed in this review.

## Introduction

Since early 1980s, adjuvant endocrine treatment involving the use of tamoxifen has shown to reduce recurrence and increase survival in estrogen receptor (ER) positive breast cancer patients (Refs 1, 2) by preventing tumour cell growth and angiogenesis, potentially via impeding the binding of estrogen to the ER or inhibiting the expression of estrogen-responsive genes (Ref. 3). For ER-positive (ER+) patients, allocation to about 5 years of adjuvant tamoxifen reduces the annual breast cancer death rate by 31% while the breast cancer mortality rate throughout the next 15 years would be approximately halved (Ref. 4). However, patients' responses to tamoxifen vary. In total, 30–50% of patients with adjuvant tamoxifen therapy experience relapse and subsequently die of the disease (Refs 2, 4).

Tamoxifen is a prodrug and extensively metabolised in the liver to more potent metabolites, including 4-hydroxy-tamoxifen and 4-hydroxy-N-desmethyl-tamoxifen (endoxifen), to elicit its pharmacological activity (Ref. 5). Heterogeneity in patients' responses to tamoxifen among breast cancer patients is consistently observed across patient populations where administration of the same dose of this drug results in a range of outcomes which include adverse events or therapeutic failure (Refs 6, 7). The complex metabolism of tamoxifen is primarily catalysed by cytochrome P450 (CYP) enzymes, amongst which CYP2D6, CYP2C19, CYP3A4, CYP2B6 and CYP2C9 are presumed to be the most important isoenzymes (Ref. 8). For example, CYP2D6 plays a pivotal role in converting tamoxifen to 4-hydroxy-tamoxifen, or converting N-desmethyl tamoxifen, the major metabolite in patients' plasma, to endoxifen. Other isoforms of CYP, including CYP3A4, CYP3A5, CYP2C9, CYP2C19 and CYP2B6, are also involved in tamoxifen metabolism.

Polymorphisms in CYPs are clinically important in the metabolism of drugs, as certain allelic variants demonstrate either altered or non-functional enzyme activity (Refs 9–11). For example, the activity of CYP2D6 enzyme is mainly determined by CYP2D6 gene, which is a polymorphic gene with more than 100 documented alleles (Ref. 12). The CYP2D6 metabolising function is associated with the variant alleles and can be grouped into four categories, including poor-metaboliser (PM), intermediate-metaboliser (IM), extensive-metaboliser (EM) and ultra-metaboliser (UM). Previous studies indicated that CYP2D6*4 is associated with non-functional enzyme activity, i.e. PM, while CYP2D6*10 and *17 are responsible for reduced enzyme activity, i.e. IM (Refs 13–15). However, the association between CYP2D6 genotype and survival is conflicting. A recent study on Mexican Mestizo patients showed that genetic phenotypes of CYP2D6 have no impact on breast cancer-free survival for patients with tamoxifen treatment (Ref. 16). Many clinical variables have been associated with drug response such as age, diet, menopausal status, lifestyle and tumour biology. Important achievements have also been obtained in optimisation of drug therapy based on the classification of diseases using protein expression profiles of the breast cancer tumours. However, a review correlating the genetic background of a patient with respect to metabolising enzymes and the patient's clinical data on survival of the therapy is still lacking. Therefore, this review aims to collate and synthesise existing evidence regarding the associative relationship between single nucleoid polymorphisms (SNPs) or genotypes of cytochrome P450 enzymes and survival in female adults with breast cancer receiving adjuvant tamoxifen treatments.

## Methods

### Search strategy

A systematic review was conducted in PubMed, EMBASE, APA PsycINFO, OVID MEDLINE and CINAHL databases by using the following keywords and Boolean logics presented in Table 1. Manual search by screening the reference lists of the included articles was conducted to identify further eligible articles.

**Table 1.** Search strategy

‘cancer’ OR ‘carcinoma’ OR ‘tumour’ OR ‘malignancy’ OR ‘neoplasm’
AND
‘breast’
AND
‘CYP2D6’ OR ‘CYP’ OR ‘cytochrome P450’
AND
‘polymorphism’ OR ‘polymorphisms’ OR ‘polymorphic’ OR ‘genetic difference’ OR ‘genetic differences’ OR ‘genotype’ OR ‘phenotype’ OR ‘genetic variations’ OR ‘genetic variants’
AND
‘survive’ OR ‘survival’ OR ‘die’ OR ‘dead’ OR ‘death’ OR ‘recurrence’ OR ‘recurrent’ OR ‘metastasis’ OR ‘relapse’

### Eligibility criteria

A study was included in the review process if it was written in English and examined the association between SNPs or genotypes of CYP450 on survival outcomes in women with diagnosed breast cancer and in adjuvant endocrine treatment. Studies were excluded if they (a) reported secondary analysis of existing evidence (review, meta-analysis), study protocol or conference abstracts; (b) qualitative study or mixed-methods study without statistical analysis; (c) reported the recurrence of breast cancer but without any types of survival rate. A full-text examination was performed to confirm their eligibility for inclusion or exclusion.

### Data extraction and analysis

The identified studies from the aforementioned databases were put in EndNote to check duplicates. After removal of the duplicates, three researchers screened the titles and abstracts of these studies based on the above inclusion and exclusion criteria. Cross check was conducted by another researcher to identify the eligibility of the studies. The full texts of eligible studies were examined by two researchers independently. Disagreements between researchers on eligibility were determined via discussion.

Further data extraction was performed by three researchers and verified by a second author after the inclusion of studies was confirmed. Data related to the study setting, participants characteristics (e.g. age, race/ethnicity), primary outcome (i.e. survival), related cytochrome P450 genes, including SNPs or genotypes of CYP450, medications for adjuvant endocrine treatment (tamoxifen or AIs), other treatments (e.g. radiotherapy), other associated factors (e.g. family history) and major findings were extracted. Any disagreements in data extraction were discussed between the reviewers to achieve unanimity.

Meta-analysis was conducted using Review Manager 5.3 software (The Cochrane Collaboration, London, United Kingdom). Hazard ratio (HR) and 95% confidence intervals (CI) for survival outcomes were synthesised, with *P* value <0.05 indicating statistically significant. Cochran's *Q* test and *I*^2^ were used to measure statistical heterogeneity. *I*^2^ statistics >50% and *P* value for Cochrane's *Q* test ⩽0.1 suggest heterogeneity, and a random-effects model was used. Otherwise, a fixed-effects model was used. The findings of the included studies have also been presented in a narrative and tabular manner for studies which did not provide HR or could not be pooled into meta-analyses.

### Assessment of reporting quality of the included studies

The study quality of the included studies was assessed using the Effective Public Health Practice Project (EPHPP), which is an appropriate quality assessment tool to encompass a variety of research designs, not only randomised controlled trial but also non-randomised trials (Ref. 17). Six components including study selection, study design, confounders, blinding, data collection methods, withdraws and drop-outs were assessed. Each component is rated strong, moderate or weak according to a standardised guide and dictionary. The global rating for the study is determined by the ratings of six components. The study is overall rated strong if there is no weak rating of the six components. Moderate rating indicates the study has only one weak rating of the six components. If two or more weak ratings were assessed on the six components, the study is rated as weak. Two researchers assessed the quality independently. If there was a discrepancy with respect to the six components ratings, the reasons were indicated in terms of the oversight, interpretation of criteria or interpretation of study. The discrepancy was solved by discussion within the research group.

## Results

### Search results

**Fig. 1.**
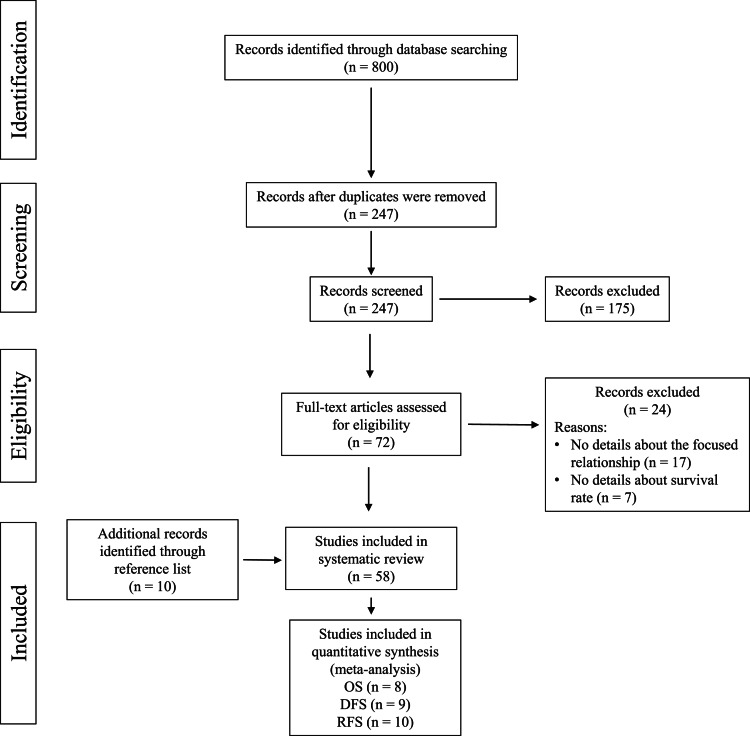
The PRISMA flow diagram.

A total of 800 records were identified with the search strategies from the above mentioned five databases. After removal of 553 duplicates, the remaining 247 citations were screened by title and abstract, among which, 175 articles were further excluded as they were not published in English and were not original articles. For the remaining 72 records, the full texts were retrieved and examined. Seventeen studies were excluded after eligibility screening because they did not provide detailed information on the focused relationship. Seven studies were excluded as they did not report the survival rate of patients with breast cancer. Nevertheless, manual search in the reference lists of the included 48 studies was conducted and 10 studies were deemed eligible for inclusion. Finally, a total of 58 studies were included in this review. Twenty-seven included studies reporting the association of CYP2D6 and survival rates were selected for meta-analysis. The Preferred Reporting Items for Systematic Reviews and Meta-Analyses (PRISMA) diagram depicting the search results is shown in Figure 1.

### Qualities of included studies

The ratings of the quality of the included studies are presented in Table 2. The reporting quality of the included studies was rated strong, moderate or weak. Only one study was rated strong, indicating that most studies had at least one weak rating for the six appraisal domains. Twenty studies were rated as moderate, all with weak rating in the blinding domain. Other included studies were rated as weak because the outcome assessor or the study participants were not blinded, or they did not report whether the outcome assessors or the study participants were aware of the intervention or research question. About one-third (*N* = 21) of the studies were rated as weak in terms of the domain of confounders. These studies failed to report the group balance at baseline with respect to confounders. Twenty-six studies were rated as weak in terms of the domain of data collection methods when assessing the reliability and validity of the assessments. Majority of included studies were rated as moderate or strong in terms of the selection bias and study design. The critical appraisal of the included studies was summarised in Table 2.

**Table 2.** Reporting quality of the included studies

Study	Selection bias	Study design	Confounders	Blinding	Data collection method	Withdrawals and dropouts	Global rating
Abraham *et al.*, 2010 (Ref. 18)	Moderate	Moderate	Weak	Weak	Weak	Moderate	Weak
Argalácsová *et al.*, 2015 (Ref. 19)	Moderate	Moderate	Strong	Weak	Strong	Not applicable	Moderate
Bijl *et al.*, 2009 (Ref. 20)	Moderate	Moderate	Weak	Weak	Strong	Moderate	Weak
Chamnanphon *et al.*, 2013 (Ref. 21)	Moderate	Moderate	Strong	Weak	Weak	Not applicable	Weak
Damkier *et al.*, 2017 (Ref. 22)	Moderate	Moderate	Weak	Weak	Weak	Strong	Weak
Damodaran *et al.*, 2012 (Ref. 23)	Moderate	Moderate	Strong	Weak	Strong	Strong	Moderate
Dezentjé *et al.*, 2013 (Ref. 24)	Moderate	Moderate	Weak	Weak	Strong	Strong	Weak
Goetz *et al.*, 2005 (Ref. 25)	Moderate	Strong	Strong	Weak	Strong	Strong	Moderate
Goetz *et al.*, 2007 (Ref. 26)	Moderate	Strong	Weak	Weak	Strong	Moderate	Weak
Goetz *et al.*, 2013 (Ref. 27)	Moderate	Strong	Weak	Weak	Weak	Weak	Weak
He *et al.*, 2019 (Ref. 28)	Moderate	Moderate	Strong	Weak	Weak	Not applicable	Weak
Helland *et al.*, 2017 (Ref. 29)	Moderate	Moderate	Strong	Weak	Strong	Not applicable	Moderate
Hertz *et al.*, 2017 (Ref. 30)	Moderate	Moderate	Weak	Weak	Strong	Not applicable	Weak
Jernström *et al.*, 2009 (Ref. 31)	Moderate	Moderate	Weak	Weak	Strong	Weak	Weak
Jorge-Aarón *et al.*, 2020 (Ref. 16)	Moderate	Moderate	Moderate	Weak	Moderate	Moderate	Moderate
Karle *et al.*, 2013 (Ref. 32)	Moderate	Moderate	Strong	Weak	Strong	Not applicable	Moderate
Kiyotani *et al.*, 2008 (Ref. 33)	Moderate	Moderate	Weak	Weak	Weak	Not applicable	Weak
Kiyotani *et al.*, 2010 (Ref. 34)	Weak	Moderate	Weak	Weak	Weak	Not applicable	Weak
Kiyotani *et al.*, 2010a (Ref. 35)	Moderate	Moderate	Weak	Weak	Strong	Not applicable	Weak
Kuo *et al.*, 2017 (Ref. 36)	Moderate	Moderate	Weak	Weak	Strong	Not applicable	Weak
Lammers *et al.*, 2010 (Ref. 37)	Moderate	Moderate	Weak	Weak	Moderate	Strong	Weak
Lan *et al.*, 2018 (Ref. 38)	Moderate	Moderate	Strong	Weak	Strong	Not applicable	Moderate
Lan *et al.*, 2018a (Ref. 39)	Moderate	Moderate	Strong	Weak	Strong	Not applicable	Moderate
Lei *et al.*, 2016 (Ref. 40)	Moderate	Moderate	Strong	Weak	Strong	Not applicable	Moderate
Malash *et al.*, 2020 (Ref. 41)	Moderate	Moderate	Strong	Weak	Strong	Not applicable	Moderate
Margolin *et al.*, 2013 (Ref. 42)	Moderate	Moderate	Weak	Weak	Strong	Not applicable	Weak
Markkula *et al.*, 2014 (Ref. 43)	Moderate	Moderate	Strong	Weak	Moderate	Strong	Moderate
Mayer *et al.*, 2019 (Ref. 44)	Moderate	Moderate	Weak	Weak	Strong	Not applicable	Weak
Moyer *et al.*, 2011 (Ref. 45)	Moderate	Moderate	Weak	Weak	Weak	Not applicable	Weak
Newman *et al.*, 2008 (Ref. 46)	Moderate	Moderate	Strong	Weak	Strong	Not applicable	Moderate
Nowell *et al.*, 2005 (Ref. 47)	Moderate	Moderate	Weak	Weak	Strong	Not applicable	Weak
Okishiro *et al.*, 2009 (Ref. 48)	Weak	Moderate	Strong	Weak	Weak	Not applicable	Weak
Park *et al.*, 2011 (Ref. 49)	Moderate	Moderate	Strong	Weak	Strong	Not applicable	Moderate
Park *et al.*, 2012 (Ref. 50)	Moderate	Moderate	Weak	Weak	Weak	Not applicable	Weak
Rae *et al.*, 2012 (Ref. 51)	Moderate	Strong	Strong	Moderate	Weak	Moderate	Moderate
Ramón y Cajal *et al.*, 2010 (Ref. 52)	Strong	Moderate	Weak	Weak	Weak	Strong	Weak
Regan *et al.*, 2012 (Ref. 53)	Moderate	Strong	Strong	Moderate	Strong	Strong	Strong
Ruiter *et al.*, 2010 (Ref. 54)	Strong	Moderate	Strong	Weak	Weak	Moderate	Weak
Saladores *et al.*, 2015 (Ref. 6)	Strong	Moderate	Weak	Weak	Weak	Strong	Weak
Sanchez-Spitman *et al.*, 2019 (Ref. 55)	Strong	Strong	Strong	Weak	Moderate	Strong	Moderate
Schroth *et al.*, 2007 (Ref. 56)	weak	Moderate	Strong	Weak	Weak	strong	Weak
Schroth *et al.*, 2009 (Ref. 57)	Weak	Moderate	Moderate	Weak	Weak	Strong	Weak
Sensorn *et al.*, 2013 (Ref. 58)	Weak	Moderate	Strong	Weak	Weak	Strong	Weak
Sensorn *et al.*, 2016 (Ref. 59)	Strong	Moderate	Strong	Weak	Moderate	Strong	Moderate
Sim *et al.*, 2018 (Ref. 60)	Weak	Moderate	Strong	Weak	Weak	Strong	Weak
Sirachainan *et al.*, 2012 (Ref. 61)	Weak	Moderate	Weak	Weak	Strong	Strong	Weak
Stingl *et al.*, 2010 (Ref. 62)	Moderate	Moderate	Strong	Weak	Strong	Strong	Moderate
Sukasem *et al.*, 2012 (Ref. 63)	Strong	Moderate	Strong	Weak	Weak	Strong	Weak
Tamura *et al.*, 2020 (Ref. 64)	Strong	Strong	Strong	Weak	Strong	Moderate	Moderate
Teh *et al.*, 2012 (Ref. 65)	Strong	Moderate	Strong	Weak	Weak	Strong	Weak
Thompson *et al.*, 2011 (Ref. 66)	Strong	Strong	Strong	Weak	Weak	Strong	Weak
Toyama *et al.*, 2009 (Ref. 67)	Strong	Moderate	Strong	Weak	Weak	Strong	Weak
Trojan *et al.*, 2013 (Ref. 68)	Weak	Moderate	Strong	Weak	Weak	Strong	Weak
Wegman *et al.*, 2005 (Ref. 69)	Weak	Strong	Strong	Weak	Weak	Strong	Weak
Wegman *et al.*, 2007 (Ref. 70)	Weak	Strong	Strong	Weak	Weak	Strong	Weak
Xu *et al.*, 2008 (Ref. 71)	Strong	Strong	Strong	Weak	Moderate	Strong	Moderate
Zeng *et al.*, 2017 (Ref. 72)	Moderate	Moderate	Strong	Weak	Moderate	Strong	Moderate
Zhang *et al.*, 2015 (Ref. 73)	Strong	Weak	Strong	Weak	Weak	Strong	Weak

### Characteristics of included studies

The characteristics of the included studies were listed in Supplementary Table S1. Fifty-eight included studies were published between 2005 and 2020 (Refs 6, 16, 18–73). The included studies have assessed different allelic variants of cytochrome P450 (CYP). Fifty-two studies reported the results on CYP2D6 gene variants (Refs 6, 16, 18–21, 23–30, 32–35, 37–44, 46–53, 55–57, 59–73). Seven studies reported on CYP2C19 variants (Refs 22, 24, 45, 48, 54, 56, 60). Two studies reported on CYP2B6 variants (Refs 24, 36). One study reported on CYP2C8 variants (Ref. 31). Two studies reported on CYP2C9 variants (Refs 24, 31). Four studies reported on CYP3A5 variants (Refs 24, 58, 59, 70). Various primary outcomes were assessed in the included studies which include breast cancer-specific survival (BCSS), breast cancer-free survival, breast cancer-specific mortality, overall survival (OS), relapse-free survival (RFS), disease-free survival (DFS), free survival time, distant disease-free survival (DDFS), progression-free survival (PFS), event-free survival (EFS), distant relapse-free survival (DRFS), cancer mortality, breast cancer mortality.

### Association of cytochrome P450 polymorphisms and survival rates in breast cancer patients

#### CYP2D6

Fifty-two included studies reported on the association of CYP2D6 polymorphisms and various types of survival rates. Among these studies, 33 studies showed positive results (Refs 6, 18–21, 23, 25–28, 32–35, 37–42, 46, 52, 56, 57, 60, 61, 63, 65, 66, 69–72) while 19 studies showed no association (Refs 16, 24, 29, 30, 43, 44, 47–50, 51, 53, 55, 59, 62, 64, 67, 68, 73). Positive correlation was reported between CYP2D6 alleles and various types of survival rates such as OS (Refs 18, 26, 32, 37, 46, 47, 56, 57), DFS (Refs 19, 21, 24, 26, 27, 38, 39, 43, 49, 52, 56, 57, 61, 63, 71), RFS (Refs 25, 26, 33–35, 48, 50, 53, 55, 66), BCSS (Refs 18, 28, 29, 42, 60), PFS (Refs 32, 62), DRFS (Refs 6, 69) based on the results of meta-analyses.

**Fig. 2.**
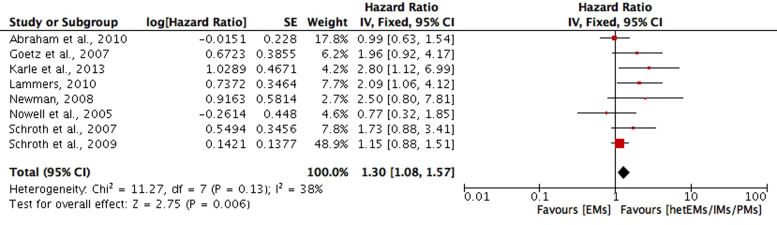
Comparative effects of CYP2D6 phenotype of hetEMs/IMs/PMs versus EMs on overall survival (OS). hetEMs, heterozygotype-extensive metabolisers; IMs, intermediate metabolisers; PMs, poor metabolisers; EMs, extensive metaboliser.

*OS*. OS in the included studies is defined as the time from registration, surgery or initiation of tamoxifen treatment to death due to any cause. Eight studies compared the OS based on CYP2D6 genotype–phenotype status (Refs 18, 26, 32, 37, 46, 47, 56, 57). The phenotype of CYP2D6 in included studies (Refs 18, 26, 32, 37, 46, 47, 56, 57) of this review was clarified into (1) poor metabolisers (PMs) for the presence of alleles of *3, *4, *5, *6, or with activity score <1; (2) intermediate metabolisers (IMs) for the presence of alleles of *9, *10, *17, *36, *41, with one null-activity allele, or with activity score between 1 and 2; (3) extensive metaboliser (EMs) if carried two functional/wild type (wt) alleles, without a CYP2D6 inhibitor, or activity score ⩾2; and (4) heterozygotype-extensive metabolisers (hetEMs) based on the presence of one null allele. Meta-analysis of the eight studies (Refs 18, 26, 32, 37, 46, 47, 56, 57) showed a significant shorter OS for decreased metabolisers (hetEMs/IMs/PMs) compared with EMs (HR = 1.30; 95% CI 1.08–1.57; *P* = 0.006; *I*^2^ = 38%) (Fig. 2). The remaining studies on OS were not included in the meta-analysis because they did not provide HR (Ref. 49) or with different comparing groups (Refs 25, 40, 41, 67, 73). In which, significant shorter OS was found in PMs compared with EMs (*P* = 0.01), and in CYP2D6*10 variant genotype (T/T) carriers compared with wild genotype (C/C) plus heterozygous genotype (C/T) carriers (*P* = 0.015) (Ref. 40). The study of Malash *et al*. also found that CYP2D6 polymorphism associated significantly with reduced OS (*P* < 0.001) (Ref. 40). However, no significant results on OS were found in CYP2D6 *4/*4 compared with *4/wt plus the wt/wt genotype (HR = 1.12, 95% CI 0.50–2.50, *P* = 0.78) (Ref. 25), or CYP2D6*10/*10 compared with wt/*10 or wt/wt (Ref. 64), or CYP2D6*10 wt genotype compared with T carriers (Ref. 73).

**Fig. 3.**
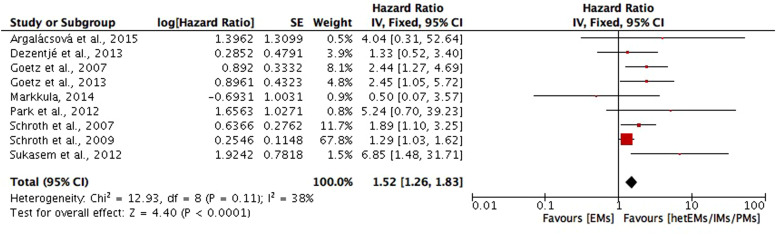
Comparative effects of CYP2D6 phenotype of hetEMs/IMs/PMs versus EMs on disease-free survival (DFS). hetEMs, heterozygotype-extensive metabolisers; IMs, intermediate metabolisers; PMs, poor metabolisers; EMs, extensive metaboliser.

*DFS*. DFS in included studies is defined as time from diagnosis, surgery, randomisation or enrolment to the following events: any recurrence (local, regional or distant) of breast cancer, contralateral breast cancer, a secondary primary breast cancer, distant metastasis or death from any cause. In which, nine studies (Refs 19, 24, 26, 27, 43, 49, 56, 57, 63) compared the DFS in CYP2D6 genotype–phenotype status of hetEMs/IMs/PMs with EMs, and the meta-analysis of the nine studies showed a significant shorter DFS in hetEMs/IMs/PMs group (HR = 1.52; 95% CI 1.26–1.83; *P* < 0.001; *I*^2^ = 38%) (Fig. 3). Studies compared the DFS between CYP2D6*10T/T with C/C plus C/T carriers (Refs 21, 38, 39, 61, 71), in which significant shorter DFS was found in T/T carriers in the study of Lan *et al*. (Ref. 38) (HR = 1.87; 95% CI 1.19–2.93; *P* = 0.006), Xu *et al*. (Ref. 71) (HR = 4.7; 95% CI 1.1–20; *P* = 0.04) and the study of Chamnanphon *et al*. (Ref. 21) (*P* = 0.046), and the study of Sirachainan *et al*. (Ref. 61) (*P* = 0.036). However, the meta-analysis was not performed due to the lack of data on HR in three studies (Refs 21, 39, 61). For other studies that reported outcomes on DFS but not included in meta-analysis because of different comparing groups, one study (Ref. 68) found no significant difference between CYP2D6 EMs with controls that had a similar distribution of characteristics (HR = 0.42; 95% CI 0.14–1.22; *P* = 0.10). One study (Ref. 25) compared CYP2D6*4/*4 with wt/wt or *4/wt genotypes, but did not found significant difference regarding DFS (HR = 1.86; 95% CI 0.91–3.82; *P* = 0.089). Similarly, no significant association with DFS was found regarding CYP2D6*10/*10, wt/*10, or wt/wt (Ref. 67). However, one study (Ref. 52) reported a significant shorter DFS in CYP2D6*4/*4, *4/*41, *1/*5 and *2/*5, compared with other CYP2D6 genotypes (*P* = 0.016).

**Fig. 4.**
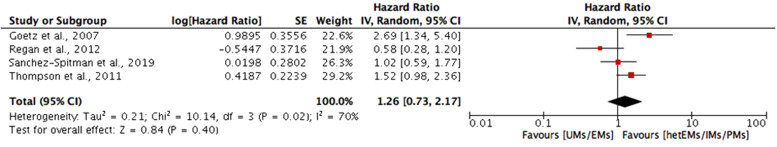
Comparative effects of CYP2D6 phenotype of hetEMs/IMs/PMs versus EMs/UMs on relapse-free survival (RFS). hetEMs, heterozygotype-extensive metabolisers; IMs, intermediate metabolisers; PMs, poor metabolisers; EMs, extensive metaboliser; UMs, ultra-rapid metabolisers.

**Fig. 5.**
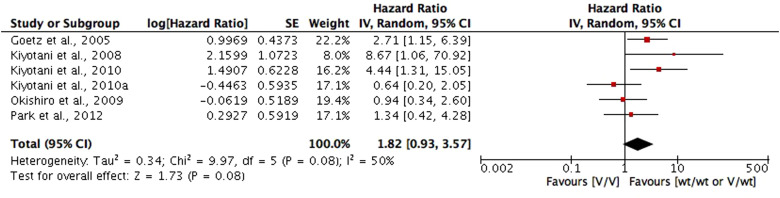
Comparative effects of CYP2D6 wt/wt or V/wt carriers versus V/V carriers on relapse-free survival (RFS). Variant genotypes (V) included CYP2D6 null alleles (*4, *5, *10, *10-*10, *14, *21, *36-*36, and *41); and wild type (wt), included *1 and *1-*1 alleles.

*RFS*. RFS in included studies is the time from surgical treatment, initiation of tamoxifen therapy, enrolment or randomisation until any recurrence of breast cancer (local, regional or distant), or a contralateral breast cancer. Among which, four studies (Refs 26, 53, 55, 66) compared the RFS between CYP2D6 genotype–phenotype status of hetEMs/IMs/PMs with ultra-rapid metabolisers (UMs)/EMs on RFS. Meta-analysis of the four studies showed a shorter RFS in hetEMs/IMs/PMs group compared with UMs/EMs group, but the difference is not significant (HR = 1.26; 95% CI 0.73–2.17; *P* = 0.40; *I*^2^ = 70%) (Fig. 4). Six studies (Refs 25, 33–35, 48, 50) compared the RFS between CYP2D6 null alleles (*4, *5, *10, *10-*10, *14, *21, *36-*36 and *41), defined as variant genotypes (V) with *1 and *1-*1 allele, defined as wild type (wt). Meta-analysis of the six studies showed a shorter RFS for V/V genotypes compared with wt/wt or V/wt genotypes, but the difference is not significant (HR = 1.82; 95% CI 0.93–3.57; *P* = 0.08; *I*^2^ = 50%) (Fig. 5). For studies not included into meta-analysis due to different comparing groups, two studies (Refs 23, 60) found a significant shorter FRS in CYP2D6 enzymatic activity <50% or CYP2D6 activity score ⩽0.5, compared with CYP2D6 enzymatic activity >50% (HR = 1.87; 95% CI 1.09–3.19) or activity score ⩾1 (HR = 7.29; 95% CI 2.92–18.17; *P* < 0.0001). One study compared CYP2D6 *4/*4 with wt/wt or *4/wt genotypes on RFS, but the difference is not significant (HR = 1.85; 95% CI 0.76–4.52; *P* = 0.176).

*BCSS*. BCSS in included studies was defined as a death in which breast cancer was given as the cause of death. Significant lower BCSS was found in CYP2D6*6b/6c genotype (HR = 1.95; 95% CI 1.12–3.40; *P* = 0.02) (Ref. 18); PMs (HR = 10) and IMs (HR = 3.3) compared with EMs (Ref. 29) or normal metabolisers (HR = 4.52; 95% CI 1.42–14.37) (Ref. 28) and in low CYP2D6 activity (Refs 42, 60).

*PFS*. PFS in included studies was defined as time from initiation of tamoxifen treatment to objective tumour progression or death; or survival time without tumour progression. One study reported a significant shorter PFS in IMs/PMs group compared with EMs groups (HR = 2.19; 95% CI 1.15–4.18; *P* = 0.017) (Ref. 32). One study also reported decreased CYP2D6 metabolisers, PMs, were associated with a shorter PFS compared with IMs/EMs (HR = 4; 95% CI 1.2–13; *P* = 0.013) (Ref. 62). However, no association between PFS and CYP2D6 genotype was found in three studies (Refs 47, 64, 72).

*DRFS*. Three studies reported DRFS, defined as time from diagnosis to the earliest occurrence of distant metastasis or death. Two studies found that improved DRFS was associated with increasing CYP2D6 activity score (HR = 0.62; 95% CI 0.43–0.91; *P* = 0.013) (Ref. 6), and that CYP2D6*4 genotype was related to decreased risk of DRFS (Ref. 69). One study did not find statistically significant difference in DRFS between CYP2D6 metabolic variants of PM and EM (HR = 0.99; 95% CI 0.48–2.08; *P* = 0.99) (Ref. 51).

Nineteen of the included studies reported that CYP2D6 genotypes were not associated with survival in breast cancer patients treated with tamoxifen (Refs 16, 24, 29, 30, 43, 44, 47–50, 51, 53, 55, 59, 62, 64, 67, 68, 73). In 11 of these studies (Refs 16, 24, 29, 30, 43, 44, 50, 51, 55, 64, 68), CYP2D6 genotyping was performed on different variants including *1, *2, *3, *4, *5, *6, *9, *10, *17, *36, *41. The patients' CYP2D6 phenotype was then determined by the results of genotyping and classified into PM, IM and EM. Statistical analyses showed that no association was found between CYP2D6 genotypes and survival outcomes such as DFS, RFS, PFS, OS and BCS. In other three included studies, negative association was found between CYP2D6*4 allele and survival rates (OS, PFS, DFS) of breast cancer patients (Refs 47, 59, 62). Other three studies also reported negative results on the association of CYP2D6*10 and survival of the breast cancer patients (OS, DFS, RFS, BCSS) (Refs 48, 67, 73).

#### CYP2C19

For the association between CYP2C19 genotypes and survival in breast cancer patients treated with tamoxifen, controversial results were also reported (Refs 22, 45, 48, 54, 56, 60). Ruiter *et al*. reported an association between CYP2C19*2 genotype and a significantly longer BCS when compared with the wild-type (HR, 0.26; 95% CI 0.08–0.87; *P* = 0.03) (Ref. 54). However, in Okishiro *et al*. (Ref. 48) and Damkier *et al*. (Ref. 22), no association was found between CYP2C19*2 and RFS or DFS, respectively. For CYP2C19*17 allelic variant, Schroth *et al*. reported that CYP2C19*17 genotype carriers had improved EFS rates (HR 0.58; 95% CI 0.32–1.01; *P* = 0.05) and long OS rate (HR 0.61; 95% CI 0.29–1.26; *P* = 0.18) (Ref. 56). However, Damkier *et al*. (Ref. 22) and Moyer *et al*. (Ref. 45) reported no association between CYP2C19*17 genotype and DFS. Similar conflicting results were also shown in combined analyses of CYP2C19 and CYP2D6 (Refs 22, 60). In Sim *et al*., the effect of genotype-predicted CYP2C19 and CYP2D6 phenotype combinations on treatment outcome was statistically significant for RFS (*P* = 0.025) as well as for BCSS (*P* = 0.026) but not for CYP2C19 alone (Ref. 60). However, in Damkier (Ref. 22), analysis of CYP2C19 genotype accounting for CYP2D6 phenotypes resulted in no significant association with DFS in breast cancer patients (Ref. 22).

#### CYP2B6

The study by Kuo *et al*. reported that CYP2B6 SNP rs3211371 was associated with poor survival for all investigated patients (*P* < 0.0001 for DDFS, DFS and OS) while it was predominantly associated with premenopausal women (*P* = 0.01 for DDFS, *P* = 0.0001 for DFS and *P* = 0.0005 for OS) but not associated with postmenopausal women (Ref. 36).

#### CYP2C8

In one of the included studies, Jernström *et al*. reported that CYP2C8*3 allele was associated with shorter DFS in patients with tamoxifen treatment (HR = 2.54; 95% CI 1.11–5.79; *P* = 0.027) (Ref. 31).

#### CYP2C9

In the studies reported by Dezentjé *et al*. (Ref. 24) and Jernström *et al*. (Ref. 31), no association was found between the CYP2C9 variants and the clinical outcome on any of the survival rates.

#### CYP3A5

Three included studies reported that the CYP3A5*3 variants were not associated with any of the survival outcomes (Refs 25, 58, 59). However, Wegman *et al*. demonstrated in the group randomised to 5 years' tamoxifen, the survival pattern shifted towards a significantly improved RFS among CYP3A5*3/*3 patients when compared to wild-type (HR = 0.2; 95% CI 0.07–0.55; *P* = 0.002) (Ref. 70).

## Discussion

Tamoxifen is a cornerstone of adjuvant breast cancer therapy. It reduces the risk of breast cancer recurrence and mortality (Refs 4, 74). Nonetheless, patients with very similar clinical characteristics can vary substantially in their clinical outcomes. Despite over 25 years of its clinical use, predictors of tamoxifen effectiveness in women remain elusive. Clinical data suggest that polymorphisms of cytochrome P450 (CYP) enzymes, which critically affect tamoxifen metabolism, result in better or worse survival rates. This review aimed to analyse the current data on the relationship between CYP genotypes and clinical outcomes on survival of the breast cancer patients.

In 58 included studies, we found that both positive and negative results were reported on the association between CYP variants and survival rates. Meta-analyses were performed in some of the included studies. In order to control the confounding effect of concomitant medications that inhibit CYP2D6, in the meta-analyses, we have adopted adjusted HR for studies that provided this value. The results of meta-analyses demonstrated that shorter OS, DFS and RFS were found in the patients with decreased metabolisers (hetEMs/IMs/PMs) when compared to EMs, though it was not significant for RFS. Many studies have focused on the relationship between CYP2D6 genotype/phenotype and tamoxifen metabolism (Refs 75,76). In individuals with intermediate and poor CYP2D6 metabolism, endoxifen concentrations have been shown to be up to 60 and 74% lower than in women with extensive CYP2D6 metabolism (Refs 11, 77, 78). Gene-dose effects have been reported for endoxifen concentrations as well as for the metabolic ratios of N-desmethyltamoxifen/endoxifen (Refs 11, 77). Women with impaired CYP2D6 metabolism and thus lower exposure to endoxifen have an unfavourable outcome compared to those with normal CYP2D6 metabolism.

Other included studies showed no significant association between polymorphisms in CYP2D6 and survival outcome on tamoxifen treatment. For example, Okishiro *et al*. (Ref. 48) reported no significant differences in RFS between breast cancer patients with CYP2D6*10/*10 genotypes and those with CYP2D6*1/*1 or CYP2D6*1/*10 genotypes, nor was there a difference between patients with CYP2C19 PM genotypes (CYP2C19*2/*2, *2/*3, or *3/*3) and those with CYP2C19 EM genotypes (CYP2C19 *1/*1, *1/*2, or *1/*3). Toyama *et al*. (Ref. 67) also demonstrated no significant correlation between patients with the CYP2D6*10/*10 genotype and survival time (DFS, BCSS and OS) when compared to those with CYP2D6 *1/*1 and *1/*10 genotypes. In the studies of Rae *et al*. (Ref. 51) and Regan *et al*. (Ref. 53), both reported a negative association between CYP2D6 metabolism genotypes and phenotypes and recurrence-free survival. In contrast, the report from Wegman *et al*. (Ref. 69) showed that patients with the CYP2D6*4/*4 genotype had statistically significant better DFS than those with heterozygous or homozygous CYP2D6*1 (*P* = 0.004 and *P* = 0.005, respectively). After analysing the included studies, the points listed below may provide some possible explanations to the varying and conflicting results.

### How many CYP genes and alleles were assessed to determine the drug metaboliser status?

The cytochrome P450 family consists of 57 genes. Many studies focused on the analysis of single P450 enzyme, most commonly on CYP2D6, or even single allele. However, apart from CYP2D6, other drug metabolising enzymes such as CYP3A5 and CYP2C19 may also influence the level of tamoxifen metabolites. Sim *et al*. (Ref. 60) reported that the effects of CYP2C19 and CYP2D6 phenotype combinations on treatment outcomes are statistically significant for RFS (*P* = 0.025) as well as for BCSS (*P* = 0.026), but not for CYP2C19 alone. This finding supports the value of combining two CYP genotypes for predicting the clinical outcomes of tamoxifen-treated breast cancer. The complex metabolism of tamoxifen, carried out by the catalytic activity of CYP2D6, CYP2C19, CYP2B6, CYP2C8, CYP2C9, CYP3A5 and other CYPs not included in this review may explain the null-association found in the studies. The formation of active tamoxifen metabolites in patients carrying reduced or increase CYP functional alleles may be sufficiently compensated through parallel and serial metabolic pathways catalysed by other P450 enzymes. Therefore, comprehensive genotyping is necessary for classifying the patients' genuine phenotypes.

### Tamoxifen treatment with or without chemotherapy

Kiyotani *et al*. (Ref. 35) reported that there was no association between CYP2D6 genotypes and RFS of the patients with the combination therapy of tamoxifen and other anticancer drugs (*P* = 0.28). In contrast, when the target was restricted only to the patients with tamoxifen monotherapy, positive association was found (*P* = 0.0002). Similar results were found when a subgroup analysis for tumour size was carried out (Ref. 35). These results suggest that the lack of association between CYP2D6 genotypes and tamoxifen response on survival may be because of the effect of concomitant drugs that were not metabolised by CYP2D6. Similar situation was reported by Stingl *et al*. (Ref. 62). The clinical outcomes for patients combined with and without chemotherapy are different from that with chemotherapy group only (Ref. 62). The absence of data on comedication available may lead to an underestimation or overestimation of the overall effect of CYP2D6 deficiency on survival rates.

In addition, plasma concentration of chemotherapeutic drugs may also be affected by drug transporters such as ATP-binding cassette (ABC) transporter family. Apart from metabolising enzymes, drug transporter polymorphisms have been addressed in an issue of tamoxifen-outcome variation. Japanese women carrying wild-type allele ABCC2 68231A > G were associated with an increased risk of recurrence during adjuvant tamoxifen therapy (Ref. 34). Homozygous CC genotype of ABCB1 3435C > T was recently shown to correlate with shorter DFS in Asian breast cancer patients treated with tamoxifen (Ref. 65). Noticeably, both studies demonstrated the significance of drug transporter genes with wild-type allele when analyses were performed in combination with impaired or absent functional variants of CYP2D6. The consistent results may reflect the important role of drug transporters in limiting intracellular drug concentration caused by defective hepatic CYP2D6.

### Use of CYP2D6 inhibitors

The concomitant use of CYP2D6 inhibitor could bias the analysis between CYP2D6 genotypes and survival rates because it could have led to different classification of CYP2D6 phenotypes. An example is selective serotonin reuptake inhibitors, which effectively reduce the incidence and severity of hot flashes in breast cancer patients (Ref. 79). Unfortunately, these antidepressant drugs inhibit CYP2D6 enzyme function, thereby reducing endoxifen plasma concentrations (Ref. 77). The effects of CYP2D6 inhibitor use on the efficacy of tamoxifen treatment have been examined in Kelly *et al*. (Ref. 77). It was found that women receiving paroxetine concurrently with tamoxifen appeared to have a higher risk of breast cancer mortality, with increases in mortality risk related to the duration of concomitant use (Ref. 77). In most of the included studies of this review, the intake of CYP2D6 inhibitors was not assessed. Only Abraham *et al*. (Ref. 18) have mentioned the result of their study that the use of CYP2D6 inhibitors did not affect BCSS or OS of the patients. The lack of data on inhibitor intake may wrongly estimate the association of CYP variants and survival rates.

### Subgroup analysis for premenopausal or postmenopausal patients

In several included studies, it was found that different results might be obtained when the analysis was done on premenopausal patients or postmenopausal patients or both cohorts. For example, Margolin *et al*. (Ref. 42) reported that in the premenopausal group of patients, there was an association between CYP2D6 activity and BCSS (*P* = 0.043). By contrast, no such association was found in postmenopausal patients. Subgroup analysis with respect to menopausal status gave indications that the overall impact of CYP2D6 genotype was derived mainly from premenopausal patients. The study by Chamnanphon *et al*. (Ref. 21) showed that CYP2D6 and CYP2C19 variants are not significantly associated with the clinical outcome in breast cancer patients taking adjuvant tamoxifen. Conversely, in a group of post-menopausal women, the polymorphisms in CYP2D6*10/*10 might be useful in predicting tamoxifen efficacy and clinical outcomes when compared to heterozygous CYP2D6*10 and homozygous wild type (CYP2D6 *1/*1). Similarly, one of the included studies by Sukasem *et al*. (Ref. 63) which was carried out in Thailand also reported that subgroup analysis in postmenopausal patients showed statistically significant inferior DFS in carriers of homozygous CYP2D6*10 genotype or IM phenotype but not in combined analysis.

The benefits from postmenopausal subgroup analysis could be at least partially explained by other studies which documented that postmenopausal breast cancer patients who are ER+ would get most benefit from receiving tamoxifen as adjuvant treatment compared to those in premenopause because the higher endogenous estrogen level might limit the tamoxifen efficacy in premenopausal patients via competitive binding to ER. Regarding the extent of ER expression in breast cancer patients, another included study by Trojan *et al*. (Ref. 68) also reported that DFS of patients with EM phenotype of CYP2D6 did not differ significantly from controls (*P* = 0.1). However, when patients with ER expression of ⩽20% were excluded from the analysis, DFS was associated with a more favourable outcome (*P* = 0.06). The result suggests a possible association between CYP2D6 phenotype and clinical outcome in ER+ breast cancer patients.

### Tamoxifen adherence

A Swedish study found that 31% of the patients who were prescribed adjuvant endocrine treatment after breast cancer surgery were non-adherent to therapy during 3 years of follow-up (Ref. 78). In the included study by Margolin *et al*. (Ref. 42), 18% of the patients had stopped tamoxifen treatment within the first year. Wegman *et al*. reported that a small subset of patients with ER+ breast cancer treated with tamoxifen who carried the CYP2D6*4 variant allele had significantly better clinical outcomes than the patients with the same variants not treated with tamoxifen (Ref. 69). Hertz *et al*. (Ref. 30) also reported conflicting results when association analyses between CYP2D6 genotypes and RFS were done in tamoxifen-treated patients or tamoxifen-untreated patients (*P* = 0.29 or *P* = 0.023, respectively). Lack of testing for tamoxifen adherence could be a limitation for data analysis. If data on tamoxifen adherence is lacking, information on drug persistence could be recorded.

In future, there is a growing interest in personalised medicine and in the use of genetic testing to provide information for selecting specific treatments for patients with cancer. Controversies exist as to whether the genetic polymorphisms of cytochrome P450 enzymes responsible for the metabolism of tamoxifen can predict breast cancer outcome in patients using adjuvant tamoxifen. A meta-analysis of data obtained from 4973 tamoxifen-treated patients with breast cancer in retrospective studies (12 globally distributed sites) suggested that strict eligibility criteria (DNA source and target alleles for CYP2D6 genotyping, menopausal status, ER status, dose of tamoxifen and periods of tamoxifen dosing) are needed to assess the CYP2D6 status and clinical outcomes in tamoxifen adjuvant therapy (Ref. 79). Limitation of relatively small numbers of patients and patient selection biases may affect the results of the study. In the study by Regan *et al*. (Ref. 53), 4861 patients have enrolled in the genotyping experiment and this study concludes a negative association between CYP2D6 genotypes and breast cancer recurrence. In addition, the association of hormones other than tamoxifen and P450 enzymes should be considered. Toremifene is an alternative to tamoxifen for adjuvant endocrine therapy in breast cancer. Lan *et al*. (Ref. 38) found that the 5-year DFS rates among different alleles of CYP2D6*10 were similar. Moreover, tamoxifen was metabolised by CYP2D6 to a significantly greater extent than Toremifene in breast tissues. Lan *et al*. (Ref. 38) reported that the genotype for CYP2D6*10 was a significant prognostic marker for DFS (*P* = 0.006). However, the CYP2D6*10 genotype was not associated with DFS in the subgroup of women who received AIs (*P* = 0.332). The results suggested that other treatments such as Toremifene or AIs might be more suitable for those groups with impaired CYP2D6 functionality than tamoxifen.

## Limitations

This review explored the association between CYP450 genetic polymorphisms and survival rates in breast cancer patients treated with adjuvant tamoxifen therapy. Despite there being almost 60 included studies in this review, the clinical significance of the pharmacogenetics to tamoxifen effectiveness remains controversial and incompletely understood. There are some limitations that need to be acknowledged: (1) Due to the heterogeneity of CYP450 genetic polymorphisms and survival outcomes examined in the studies, meta-analysis could not be performed in all included studies. (2) No comparison of tamoxifen and aromatase inhibitors (another hormone commonly used in adjuvant endocrine therapy) on the clinical outcomes was done by authors in or of the included studies. The advantage and disadvantage of tamoxifen over aromatase inhibitors cannot be concluded. (3) Genetic polymorphism of CYP450 is influenced by ethnicity. Different ethnic groups involved in different studies may limit the generalisability of the findings. (4) Limited sample size especially for the genotyping from tissues which may limit the detection of true effects.

## Conclusions

At this stage, controversies still exist on the association of CYP450 genotypes and survival outcome by tamoxifen treatment in breast cancer patients. Comprehensive genotyping including various CYP450 enzymes should be considered, and the genotyping results need to be interpreted in a clinical setting where tamoxifen adherence, menopausal status, CYP450 inhibitors and chemotherapeutic drugs may have a significant influence on the outcomes. More studies are still needed to come to a conclusion if it is beneficial or not for breast cancer patients to have CYP450 genotyping before considering adjuvant tamoxifen therapy.

## Supplementary material

For supplementary material accompanying this paper visit http://doi.org/10.1017/erm.2021.28. click here to view supplementary material
