# Supplementary material for: Association between genetic polymorphisms in cytochrome P450 enzymes and survivals in women with breast cancer receiving adjuvant endocrine therapy: a systematic review and meta-analysis
Source: Expert Rev Mol Med. 2022 Jan 7;24:e1. doi: 10.1017/erm.2021.28 (PMC9884795; doi:10.1017/erm.2021.28)
Supplement: Supplementary file 1 [file S1462399421000284sup001.docx]

**Supplementary table S1 The characteristics of the included studies**

| Study | Study settings | Participant characteristics | Study design &methods | Primary outcome variable | Cytochrome P450 gene(s) examined | Drugs for adjuvant treatment | Other associative variables | Major findings |
| --- | --- | --- | --- | --- | --- | --- | --- | --- |
| Abraham et al., 2010 [18] | Cases from Studies of Epidemiology and Risk factors in Cancer Heredity (SEARCH) and ascertained through the Eastern Cancer Registration and Information Centre (ECRIC) in England,  Cambridge, UK | - - - - Women with invasive breast cancer (N=6,640 cases): - WITH self-reported tamoxifen treatment (N=3155) - WITHOUT self-reported tamoxifen treatment (N=3485)   - - - Age: 23 - 69 years       - Median age: 53       - Caucasian ethnicity (98.8%) | - Population based case-cohort study - Active follow-up at three and five years after diagnosis and then at five-year intervals until the end of 2005. - The association between genotype, OS and BCSS was evaluated using Cox proportional hazards regression analysis. | BCSS, OS | CYP2D6*4, *5, *6, *41, *9, *10 | Tamoxifen | Tumour morphology, grade, and stage, concomitant use of drugs, ER status, surgery, chemotherapy | - There was no statistically significant association between BCSS or OS and genotype for any of the tagSNPs for common variation at the CYP2D6 locus either in patients receiving or not receiving tamoxifen. - In tamoxifen treated patients, there was weak evidence that the poor-metaboliser variant, CYP2D6*6 (MAF=0.01), was associated with decreased BCSS (HR=1.95; 95% CI: 1.12-3.40; *P*=0.02). - There was no significant association between BCSS or OS and the putative functional alleles termed CYP2D6*1; *4; *5; *9; *10; *41; *UM. - There was no difference in survival between PM/IM and EM (BCSS: HR=0.93; 95% CI: 0.55 to 1.57; *P*=0.78 and OS: HR=0.98; 95% CI: 0.63 to 1.54; *P*=0.94). |
| Argalácsová et al., 2015 [19] | Department of Oncology, General Teaching Hospital in Prague, Czech Republic | - Premenopausal patients with breast cancer indicated to 5 years adjuvant tamoxifen treatment with or without adjuvant chemotherapy (N=71) - Age: 26 to 52 years - Ethnicity/race: Not provided | - Retrospective study - Timeframe for data collection: 1985-2011 - Timeframe for data analysis: Not mentioned. - Statistical analysis method: Kruskal-Wallis test, Pearson's $\chi^{2}$ test and Mantel-Cox, Kaplan-Meier and Cox proportional-hazards model | DFS | CYP2D6*1, *3, *4, *5, *6, *7, *8, *9, *10, *11, *14, *15, *17, *19, *20, *25, *26, *29, *30, *31, *35, *36, *40, *41 and gene copy | Tamoxifen, chemotherapy | Tumour stage, nodal status, histology, grade, PR status, Her status, chemotherapy, radiotherapy, local therapy, rs1045642, rs2032582 | - For the CYP2D6 polymorphism, PM phenotype resulted in worse outcome in comparison to EM subjects with HR of 4.04 (95% CI: 0.31-52.19). - Patients using CYP2D6 inhibitors had non-significantly shorter time-to-event as compared to never users resulting in hazard ratio of 2.06 (95% CI: 0.40-10.63). |
| Bijl et al., 2009 [20] | Inhabitants  aged 55 years and older of one district of Rotterdam  (Ommoord) | - Patients aged 55 years or older and used tamoxifen at any time during the study period. (N=85) - Mean age: (first tamoxifen use): 75.5 - Ethnicity/race: Not provided | - Population-based cohort study - Timeframe for data collection: Not mentioned - Timeframe for data analysis: Not mentioned - Statistical analysis method of associative relationship: Cox proportional hazard models | Cancer mortality, breast cancer mortality | CYP2D6*4 | Tamoxifen | Age, CYP2D6 inhibitors, tamoxifen duration, dose and calendar time | - Breast cancer mortality was significantly increased in patients with the *4/*4 genotype (HR=4.1, CI 95%: 1.1–15.9, *P*=0.041) compared to wild type patients. The breast cancer mortality increased with a hazard ratio of 2.0 (CI 95%: 1.1–3.4, *P*=0.015) with each additional variant allele. - No increased risk of all- cause mortality or all-cancer mortality was found in tamoxifen users carrying a CYP2D6*4 allele. - The risk of breast cancer mortality is increased in tamoxifen users with decreased CYP2D6 activity |
| Chamnanphon et al., 2013 [21] | Department of Medicine, Ramathibodi Hospital in Bangkok, Thailand. | - Pre- and postmenopausal women (N=57) - Age: 48.9 - 10.6 (Mean, SD) years - Ethnicity/race: Not provided | - Retrospective study - Timeframe for data collection: February 1997 to January 2008 - Timeframe for data analysis: Not mentioned - Statistical analysis method of associative relationship: The univariate Cox proportion hazard model | DFS | CYP2D6, CYP2C19 | Tamoxifen | Age, menstrual status, tumour size, ER and PR status, Her-2, grading, lymph node status, LVI, margin, chemotherapy, radiation | - Kaplan–Meier estimates showed significantly shorter DFS in patients with homozygous TT when compared to those with heterozygous CT or homozygous CC at nucleotides 100C.T and 1039C.T (CYP2D6*10) in post-menopausal patients (log-rank test; *P*=0.046). |
| Damkier et al., 2017 [22] | 12 sites representing 9 countries (data from the large publicly available International Tamoxifen Pharmacogenomics Consortium (ITPC) dataset) | - Pre- and postmenopausal women (N=2102) - Tamoxifen 20 mg/day - Age: 59 - 13 (Mean, SD) years - Ethnicity/race: not provided | - Data from the large publicly available International Tamoxifen Pharmacogenomics Consortium (ITPC) dataset - Timeframe for data collection: Not mentioned. - Timeframe for data analysis: Not mentioned - Statistical analysis method of associative relationship: Cox regression | DFS | CYP2C19*1, *2, *17 | Tamoxifen, radiation, chemotherapy | Age, ethnicity, menopausal status, tumour grade and stage, PR status, other adjuvant therapy (radiation and chemotherapy) | - For the CYP2C19*2 allele, the HR was 1.05 (95% CI: 0.78–1.42) and 0.79 (95% CI: 0.32–1.94) for hetero- and homozygote carriers, respectively. - The corresponding HR for hetero- and homozygote carriers of the CYP2C19*17 allele were 1.02 (95% CI: 0.71–1.46) and 0.57 (95% CI: 0.26–1.24), respectively. Accounting for CYP2D6 genotype status did not change these estimates. |
| Damodaran et al., 2012 [23] | In a tertiary care hospital in South India | - Breast cancer patients receiving adjuvant tamoxifen therapy at present or had completed 5 years of tamoxifen (N=132) - Age: recurrence 51.32 - 10.99; no recurrence 50.41 - 8.89 (Mean, SD) years - Ethnicity/race: Not provided | - Longitudinal cohort study. - Timeframe for data collection: April 2010 to July 2011 - Timeframe for data analysis: Not mentioned. - Statistical analysis method of associative relationship: Chi-square test, Fisher's exact test and Cox proportional hazards model | RFS | CYP2D6 alleles *1, *2, *4, *5, *10. | Tamoxifen | Tumour size, nodal status, metastasis, ER and PR status, chemotherapy and radiotherapy, hot flashes | - Reduced CYP2D6 activity is associated with poor treatment outcomes, in terms of increased risk of recurrence and shorter RFS, in breast cancer patients on adjuvant tamoxifen therapy. |
| Dezentjé et al., 2013 [24] | Hospital in Dutch and from the Tamoxifen Exemestane Adjuvant Multinational (TEAM) trial | - Postmenopausal women with breast cancer (N=731) - Age: 66.1 - 9.2 (Mean, SD) years - Ethnicity/race: Not provided | - Longitudinal cohort study. - Timeframe for data collection: Not mentioned. - Timeframe for data analysis: Not mentioned - Statistical analysis method of associative relationship: Correlational analysis and cox regression analysis | DFS, DFS on tamoxifen (DFS-t) | CYP2D6*3, *4, *6, *14, *41  CYP2C9*2, *3  CYP2C19*2, *17  CYP2B6*6, *8  CYP3A5*3 | Tamoxifen | Age, type of surgery, tumour stage, nodal stage, adjuvant radiotherapy, concomitant use of CYP2D6 inhibitors | - No association was found between the CYP2D6 genotype or predicted phenotype and DFS-t (poor vs. extensive metabolizers: unadjusted HR: 1.33, 95% CI: 0.52–3.43; *P*=0.55). - In postmenopausal early breast cancer patients treated with adjuvant tamoxifen followed by exemestane, neither CYP2D6 genotype nor phenotype did affect DFS-t. |
| Goetz et al., 2005 [25] | Not provided  USA | - Postmenopausal women with resected ER+ breast cancer (N=256) - Age: 73 (56-87) in CYP2D6*4/*4 group - Race: 100% white in CYP2D6*4/*4 group | - Retrospective study - Timeframe for data collection: Not mentioned - Timeframe for data analysis: Not mentioned - Statistical analysis method of associative relationship: Kaplan-Meier estimates, the log-rank test, generalized Wilcoxon test. | RFS, DFS, OS | CYP2D6 | Tamoxifen | Nodal status and tumour size, age, extent of surgery, primary tumour size, axillary lymph node status, and ER status | - Women with the CYP2D6*4/*4 genotype had significantly worse RF-time and DFS but not OS compared with either the *4/wt or the wt/wt genotype (log-rank *P*=0.030, *P*=0.020, and *P*=0.360, respectively). - Neither RF-time, DFS, nor OS was found to differ in terms of CYP3A5*3 genotype (A/A v A/G v G/G; log-rank *P*=0.854, 0.937, and 0.950 respectively). |
| Goetz et al., 2007 [26] | Not provided  USA | - Postmenopausal women with resected ER+ breast cancer (N=256) - Age: 68 (Mean, ranged 42-87) - Ethnicity/race: Caucasian (95%), Afro-American (1%), Native American (1%) and others (3%) | - Retrospective study - Timeframe for data collection: Not mentioned. - Timeframe for data analysis: Not mentioned - Statistical analysis method of associative relationship: Kaplan-Meier estimates, the log-rank test, and univariate Cox proportional hazard models | RFS, DFS, OS | CYP2D6 | Tamoxifen | Tumour size greater than 3 cm and positive nodes | - Patients with decreased metabolism had significantly worse RFS (adj HR=1.74, 95% CI: 1.10-2.74; *P*=0.017), DFS (long-rank *P*=0.082), compared with patients with extensive CYP2D6 metabolism. - Compared with EM, PM had the most significant risk of breast cancer relapse (HR: 3.12, *P*=0.007), RFS (*P*=0.005), DFS (*P*=0.008), and worse OS (*P*=0.077) - IM did not have shorter TTBR (*P*=0.338) but tended to have worse RFS (*P*=0.075) and DFS (*P*=0.097), compared to EM. |
| Goetz et al., 2013 [27] | Austrian Breast and Colorectal Study 8 (ABCSG trial 8) | - early stage breast cancer patients within 6 weeks after surgery to either 5 years of tamoxifen (Arm A) or to 2 years of tamoxifen followed by anastrozole for 3 years (Arm B) - Arm A: N=453, Arm B: N=423 - Age: 49-80, mean 68 (cases), 47-80, mean 66 (controls) - Race: Not mentioned | - Case-control study - Timeframe for data collection: Not mentioned. - Timeframe for data analysis: Not mentioned - Statistical analysis: Conditional logistic regression modeling (CLRM) | DFS | CYP2D6 | Tamoxifen or tamoxifen and then anastrozole | Tumour stage, nodal status, Her2 status | - In Arm A: PM/PM (OR: 2.45, 95% CI: 1.05–5.73; *P*=0.04) had a higher odds of a disease event relative to EM/EM.   In Arm B: no significant association was found between CYP2D6 genotype and disease event   - Secondary analysis: non-significant higher odds of a disease event was observed for PM/PM relative to EM/EM in the first 2 years of tamoxifen for both arms: Arm A: OR = 2.54, *P*=0.25 and Arm B: OR = 2.60, *P*=0.46. During years 3–5, in Arm A, PM/PM (OR 2.40, 95% CI: 0.86 to 6.66, *P*=0.09) and IM/PM or EM/PM (OR 1.70, 95% CI: 0.91–3.17, *P*=0.09) had a trend towards an increased odds of a disease event relative to EM/EM. |
| He et al., 2019 [28] | Stockholm-Gotland Breast Cancer Register and  Swedish Prescribed Drug Register, Sweden | - Patients diagnosed with stages I to III breast cancer between 2005 and 2012 - LIBRO1 cohort (N=950)   KARMA cohort (N=551)   - Median age at cancer diagnosis was 58.1 years | - Cohort study - Timeframe for data collection: 2005-2012 - Timeframe for data analysis: Not mentioned. - Statistical analysis method of associative relationship: delayed-entry Cox regression analysis | BCSM | CYP2D6 | Tamoxifen | Symptom-relieving drugs, tamoxifen treatment discontinuation | - Ultrarapid CYP2D6 metabolizers had a significantly higher breast cancer–specific mortality (adjusted, HR: 4.52; 95% CI: 1.42-14.37) compared with normal metabolizers. - When comparing poor CYP2D6 metabolizers to normal metabolizers, a more than twofold increased breast cancer–specific mortality rate was seen (adj. HR: 2.59; 95% CI: 1.01 to 6.67). |
| Helland et al., 2017 [29] | Oslo, Norway | - Breast cancer patients with hormone receptor positive tumours received tamoxifen therapy (N=99) - Age: 56 (Mean, ranged 34-78) - Race: Not mentioned | - Retrospective observational study - Timeframe for data collection: May 1995 to December 1997 - Timeframe for data analysis: Not mentioned. - Statistical analysis method of associative relationship: log-rank test | BCSS, OS | CYP2D6 | Tamoxifen | Tumour size, nodal status, and chemotherapy | - Patients with low concentrations of z-4-hydroxy-tamoxifen had BCSS of 60% compared to 84% in patients with z-4OHtam concentrations > 3.26 nM (*P*=0.020, log-rank hazard ratio=3.56, 95%CI=1.14-11.07). - Patients with Z-4-hydroxy-N-desmethyl-tamoxifen levels ≤ 9.00 nM BCSS was 57% compared to 84% for patients with concentrations > 9.0 nM (HR=3.73, 95%CI=1.05-13.22; *P*=0.029). - Low concentrations of Z-4OHtam and Z-endoxifen were associated with poorer survival after adjusting for clinically relevant variables (HR=4.27, 95%CI 1.35-13.58, and HR=3.70, 95%CI=1.03-113.25, respectively) - Overall survival analysis showed similar survival differences for both active metabolites. |
| Hertz et al., 2017 [30] | Secondary analysis from two breast cancer databases maintained by the breast center at Baylor College of Medicine, Houston, USA | - Early breast cancer patients with surgery and tamoxifen (N=476) vs. no systematic treatment (N=481) - Age (median): 67.0 in tamoxifen treated group, 66.0 in untreated group - Ethnicity/race: United States | - Retrospective cohort study - Timeframe for data collection: secondary analysis using patients from breast cancer databases and biobanks, recruited patients from 1970 to 1999 - Timeframe for data analysis: Not mentioned - Statistical analysis method of associative relationship: Chi square and fisher's exact tests, and log-rank test | RFS | CYP2D6 | Tamoxifen | Age, tumour size, positive nodes. | - CYP2D6 AS was not associated with RFS in tamoxifen treated patients in univariate analyses (*P*>0.2), but increasing AS was associated with better RFS in adjusted analyses (HR=1.43; 95%CI: 1.00-2.04, *P*=0.05). - For patients that did not receive tamoxifen treatment, increasing CYP2D6 AS, and AS >0, were associated with superior RFS |
| Jernström et al., 2009 [31] | The Lund University Hospital and The Helsingborg Hospital, Sweden | - Women with first breast cancer (N=652) - Age: median is 59.6 and IQR is 50.7-67.0 - Ethnicity/race: Sweden | - Prospective study with pre- and post-test design. - Timeframe for data collection: October 2002 to October 2007 - Timeframe for data analysis: Not mentioned. - Statistical analysis method of associative relationship: Kaplan-Meier log-rank test and Cox regression models | BCFS | CYP2C8*3  CYP2C8*4  CYP2C9*2  CYP2C9*3 | Tamoxifen, oral contraceptive, hormone replacement therapy | Age, tumour size, axillary lymph node status, progesterone receptor status, histological grade, and other haplotypes | - The increasing number of CYP2C8*3/*1/*2/*1 alleles was associated with shorter DFS in patients with invasive tumours who had been treated with tamoxifen prior to the last follow-up or event (log rank 6.36; 1 df; *P*=0.012). |
| Jorge-Aarón et al., 2020 [16] | Oncology department of the specialty medical unit and the primary-care clinics of the Mexican social security Institute in Merida, Yucatan, Mexico | - Patients (N=71) with breast cancer undertaking adjuvant tamoxifen treatment (20 mg/day) - Age: 50.7 ± 11.4 - Ethnicity/race: Mexican Mestizo | - Retrospective cohort study - Timeframe for data collection: January 2013 to March 2015 - Timeframe for data analysis: Not mentioned. - Statistical analysis method of associative relationship: Odds ratios | BCFS | CYP2D6 | Tamoxifen, contraceptive therapy, chemotherapy, radiotherapy | Premenopausal or postmenopausal | - No differences were found between groups according to CYP2D6 GP (gNM + gUM vs gPM + gIM) and BCFS rates (P=0.45). |
| Karle et al., 2013 [32] | Palliative setting | - Advanced breast cancer patients treated with tamoxifen (20 mg per day for >30 days) (N=88) (EM group N=75; IM or PM group N=13) - Age: 59 (35-90) (Median age and range in years) in EM group; 56 (29-72) (Median age and range in years) in IM or PM group | - Cohort study - At the beginning of the first Tamoxifen prescription for metastatic disease until their death or until the end of the study period. - Kaplan-Meier product-limit method, generalized Wilcoxon test, Cox proportional hazards model andχ^2^ test | PFS, OS | CYP2D6 | Tamoxifen | Metastatic sits, age, and adjuvant hormonal therapy | - IM + PM had a signiﬁcant shorter PFS compared to patients with EM. (PFS: HR=2.19; 95% CI: 1.15–4.18; *P*=0.017). After adjusting by metastatic sites, number of metastatic sites, age, and adjuvant hormonal therapy (PFS: HR=2.14; 95% CI: 1.11–4.13; *P*=0.023). - OS was signiﬁcantly shorter for patients with IM +PM compared to EM-group (OS: HR=2.79; 95% CI: 1.12–6.99; *P*=0.028). - The OS rate at 5 years was 76.3% (95% CI: 64.5% -88.0%) for the EM-group and 45.8% (95% CI: 11.5% -80.2%) for the IM+PM-group. - There was no signiﬁcant difference in OS for patients with clinical beneﬁt stratiﬁed by the EM and IM+PM phenotype groups - There were 46.2% of the patients with an IM or PM phenotype had a disease progression in comparison with 16% of the EM (Pearson χ^2^ *P*=0.013). |
| Kiyotani et al., 2008 [33] | Tokushima Breast Care Clinic | - Invasive breast cancer patients received surgical treatment and adjuvant monotherapy of tamoxifen at a dose of 20 mg/body/day for 5 years. (N=67) - Age at surgery (years): median: 50; range: 34-82 - For nodal status, about 71.6% were n0 and 28.4% were n1. About 4.5% were HER2 positive and 71.6% were HER2 negative | - Cohort study - Timeframe for data analysis: September to November in 2007 - Cochran-Armitage test; Kaplan–Meier methods; log-rank test; Cox proportional hazard analysis | RFS | CYP2D6*4, *5, *6, *10, *14, *18, *21, *41 | Tamoxifen | Tumour size | - Kaplan–Meier estimates indicated the signiﬁcantly shorter RFS for patients with CYP2D6*10/*10 than those with CYP2D6*1/*1 or those with CYP2D6*1/*1 + *1/*10 (*P*=0.0031 or *P*=0.0010). - In the univariate Cox proportional hazard analysis for RFS, CYP2D6 genotype (*10/*10 versus *1/*1) and tumour size were considered to be signiﬁcantly associated factors. |
| Kiyotani et al., 2010 [34] | Shikoku-*10 collaborative group | - Primary breast cancer patients received tamoxifen 20 mg/d for 5 years (N=282) - Age: Median: 51; range: 31-83 - About 81.6% were negative nodal status and 17.0% were positive nodal status. About 1.8% were HER2 positive and 34.4%were HER2 negative | - Cohort study - Timeframe for data collection: September 2007 to April 2009 - Kaplan-Meier method; Cox proportional hazards analysis | RFS | CYP2D6:  *V*: *4, *5, *10, *10-*10, *14, *21, *36-*36, and *41.  *wt*: *1 and *1-*1 | Tamoxifen | Tumour size and nodal status | - Patients carrying one or two variant alleles (*wt*/V or *V/V*) had signiﬁcantly shorter RFS compared with patients with homozygous wild-type alleles (*wt/wt*; log-rank *P*=0.0002). - CYP2D6 genotype was an independent indicator of RFS after adjustment of tumour size and nodal status (trend *P*=0.000036). The adjusted HRs of patients with *wt/V* and *V/V* genotypes, relative to patients with *wt*/*wt*, were 4.44 (95% CI: 1.31-15.00) and 9.52 (95% CI: 2.79-32.45), respectively. |
| Kiyotani et al., 2010a [35] | Not mentioned | - Primary breast cancer patients treated with tamoxifen-combined therapy (N=167) - Age: 47 (Median), ranges from 35 to 73 | - Retrospective cohort study - Timeframe for data collection/analysis: not mentioned - Statistical analysis methods: not mentioned | RFS | CYP2D6 | Tamoxifen and concomitant treatment | Tumour size and nodal status. | - No significant association between the CYP2D6 genotype and the RFS of the patients with the combination therapy of tamoxifen and other drugs (trend log-rank P = 0.28). - The HRs for RFS of patients with wt/V and V/V were 1.05 (95% CI: 0.48–2.27, *P*=0.91) and 0.64 (95% CI: 0.20–1.99, *P*=0.44) in the patients with the tamoxifen and concomitant drugs compared with the wt/wt genotype. |
| Kuo et al., 2017 [36] | National Taiwan University Hospital | - Newly diagnosed patients with stage I or II hormone receptor-positive early breast cancers (N=414) - Age: 48 years (median), 23-81 years (range) - In 414 patients, 349 (84.3) were Infiltrating ductal carcinoma, 16 (3.9) were Infiltrating lobular carcinoma. - Grade: I: 171 (41.3%); II: 187 (45.2%); III: 56 (13.5%) | - Cohort study - Timeframe for data collection: January 1, 1994 and June 30, 2006. - Stepwise selection Cox model | DDFS, DFS, OS | CYP2B6  SNPs: rs3211371; rs4802101; rs3211371 | Tamoxifen; ovarian ablation or luteinizing hormone-releasing hormone | Age, lymph node, menopausal status, pathology, grade, tumour size, hormone receptor status, adjuvant hormone therapy, chemotherapy | - CYP2B6 rs3211371 (T/C vs. C/C) were associated with poor survival for all women; and was predominantly associated with premenopausal women. - Patients with CYP2B6 rs3211371 (T/C) had significantly poorer DDFS, DFS and OS than those with CYP2B6 rs3211371 (C/C). - CYP2B6 rs3211371 (T/C vs. C/C) was associated with poor prognosis for women receiving adjuvant hormonal therapy along without adjuvant chemotherapy (adj. HR= 68.6, 95% CI: 6.7-697.4, *P*=0.0004 for DDFS; adj. HR=126.5, 95%CI: 7.9-2022.4, *P*=0.0006 for DFS, and adj. HR=297.3, 95%CI: 16.3-5420.9, *P*=0.0001 for OS). |
| Lammers et al., 2010 [37] | The Daniel den Hoed cancer centre of the Erasmus MC  University hospital in The Netherlands | - Patients with metastatic breast cancer, ER+ and/or PR+, receiving a tamoxifen dose of 40 mg per day - Enrolled patients (N=116) - Genotyped patients (N=99) | - Cohort study - Timeframe for data collection: 2000-2008 - Statistical analysis methods: Kaplan–Meier and log-rank test | OS | CYP2D6*3, *4, *5, *6, *10, *41 | Tamoxifen | Age at start of  tamoxifen therapy for metastatic disease, race, ER and PR status,  treatments before tamoxifen therapy, number and location of  metastatic sites, CYP2D6-inhibiting co-medication | - OS in EMs and IMs was not significantly different (HR=0.87; 95% CI: 0.50–1.50; *P*=0.62). - OS was significantly shorter for the PMs compared with EMs (HR=2.09; 95% CI: 1.06–4.12; *P*=0.034). - OS-time period for PMs was 5.0 years (95% CI: 4.1–5.9) compared with 7.9 years (95% CI: 6.2–9.5) for the other predicted phenotype groups, which is statistically significantly different (*P*=0.012). |
| Lan et al., 2018 [38] | National Cancer Center in China | - Patients with primary breast cancer who received adjuvant TAM (N=325) or AIs (N=453, - In TAM treatment group: - C/C genotype (N=101); Age: ≥50: N=27; <50: N=74 - C/T genotype (N=153); Age: ≥50: N=35; <50: N=118 - T/T genotype (N=71); Age: ≥50: N=21; <50: N=50 | - Cohort study - Timeframe for data collection: June 1991 to March 2014 - Method; Kaplan-Meier, Cox regression model | DFS | CYP2D6*10 C/C, C/T and T/T | Tamoxifen or aromatase inhibitors | Age, grade, T stage, N stage, adjuvant chemotherapy, adjuvant radiotherapy, and ER, PR and C-erbB2 status | - Women with T/T genotype had a lower 5-year DFS rate than women with wild-type C/C or heterozygous C/T genotype (54.9% vs. 70.9%, *P*=0.007). - The 5-year DFS rate in patients with C/T was similar to patients with C/C in TAM patients (70.6% vs. 71.3%, *P*=0.833). - The T/T genotype remained an independent prognostic marker of DFS in multivariate analysis compared to the C/T plus C/C genotype (HR=1.87; 95% CI: 1.19–2.93; *P*=0.006). - The CYP2D6*10 genotype was not signiﬁcantly associated with DFS in the subgroup of women who received AIs (*P*=0.332). |
| Lan et al., 2018a [39] | National Cancer Center in China | - Patients with adjuvant Toremifene (TOR) treatment after completion of primary surgery (N=115). Age: ≥50: N=12; <50: N=103. - Breast cancer patients with adjuvant tamoxifen (N=115). TAM group: ≥50: N=20; <50: N=95. - Clinical stage: - TOR group: I: N=43; II: N=53; III; N=12; unknown: N=7. - TAM group: I: N=28; II: N=64; III; N=17; unknown: N=6. | - Cohort study - Timeframe for data collection: TOR group: January 2001 to December 2012. TAM group: June 1991 to March 2014. - Kaplan–Meier method; Cox regression model | DFS | CYP2D6*10 C/C, C/T and T/T | Toremifene, tamoxifen | Clinical stage and HER-2 status | - CYP2D6 *10 genotype was not signiﬁcantly associated with DFS in TOR group. The 5-year DFS rates among the CYP2D6 *10 C/C, C/T and T/T groups were similar (87.5% vs. 90.6% vs. 90.9%, *P*=0.737). - Patients with the CYP2D6 *10 T/T genotype had a lower 5-year DFS rate than those with the C/C or C/T genotype in TAM group (*P*=0.003). - For all of the 50 CYP2D6 *10 T/T genotype patients, patients receiving TOR treatment had a signiﬁcantly higher 5-year DFS rate than patients receiving TAM (90.9% vs. 67.9%, *P*=0.031). - For all of the remaining 170 CYP2D6 *10 C/C or C/T genotype patients, no signiﬁcant difference in 5-year DFS rates (89.2% vs. 85.1%, *P*=0.188). |
| Lei et al., 2016 [40] | Zhejiang Provincial Cancer Hospital | - Breast cancer patients who received or receiving TAM as adjuvant endocrine therapy after surgery (N=72: C/C group N=18, T/T group N=23, C/T group N=31) - Age (mean±SD): C/C group: 42.11±5.35; T/T group: 43.96±8.65; C/T group: 45.68±7.40. - Clinical stage: C/C group: I (N=3); II (N=10); III (N=5). T/T group: I (N=7); II (N=9); III (N=7). C/T group: I (N=8); II (N=15); III (N=8) | - Cohort study - Timeframe for data collection: January 1993 to October 2008 - Cox proportional hazards regression model | DFS, OS | CYP2D6*10  C/C, T/T, C/T | Tamoxifen | Diagnosed age, number of lymph node metastasis, ER/PR/HER-2 status, menopause, hot flashes and genotype-grouping, tumour size and grades | - The CYP2D6 C100T genotype was significantly associated with DFS and OS in the subgroup of patients below 40 years of age. T/T carriers had shorter DFS and OS than C/C and C/T carriers (*P*=0.015 for both DFS and OS). - C/C, C/T and T/T carriers were not prognostic factors for DFS (B=0.333, *P*=0.409) and OS (B=-0.109, *P*=0.663) in TAM treated breast cancer patients. |
| Malash et al., 2020 [41] | National Cancer Institute, Cairo University, Cairo, Egypt. | - Patients with metastatic breast cancer (N=157) - Age: 51.63 (10.5) years and the median was 52 years. - Ethnicity/race: Egyptian | - Prospective cohort study - Timeframe for data analysis/collection: from January 2014 to December 2016. - Statistical analysis method of associative relationship: $\chi$^2^ tests, Fisher's exact, and log rank test. | OS | CYP2D6*3, *4, *10, *41 | Tamoxifen | PR status, age, HER2-neuo protein expression | - Significant association between CYP2D6 polymorphism and reduced OS in both responder and refractory groups to tamoxifen treatment (*P*<0.001). |
| Margolin et al., 2013 [42] | Oncology Department of Huddinge University Hospital and Södersjukhuset in Stockholm, Sweden | - Newly diagnosed invasive breast cancer with tamoxifen treatment (N=385) - Age: 60.2 (mean), ranged 30-88 - Tumour stage: T1: 73.8%; T2: 23.3%; T3: 2.2%; T4: 0.6% - Death: in breast cancer: 10.5%; Other cause: 11.2% | - Cohort study - Timeframe for data collection: November 1998 to May 2000 - Log-rank test and multivariable Cox proportional hazard models, Kaplan–Meier method | BCSS | CYP2D6 | Tamoxifen | Age at onset, tumour size, lymph node involvement, grade, ER status, adjuvant chemotherapy, and concomitant CYP2D6 inhibitors | - There was an association between low CYP2D6 activity (≤50% of normal) with BCSM (*P*=0.034). - A stepwise decrease in CYP2D6 activity was closely associated with increased risk of breast cancer recurrence and BCSM. - In the premenopausal group of patients, there was an association between CYP2D6 activity and recurrence (*P*=0.014) as well as BCSS (*P*=0.043). - When including all 382 patients, CYP2D6 activity had no statistically significant influence on breast cancer recurrence or BCSS. |
| Markkula et al., 2014 [43] | Department of Pathology at Skane University Hospital in Lund, Lund University, Sweden | - Tamoxifen-treated breast cancer patients with ER+ tumours (N=333) | - Cohort study - Timeframe for data collection: 2002-2008 - Statistical analysis method: Kaplan-Meier and Cox regression models | BCFS | CYP2D6*3, *4, *6, *10, *41 | Tamoxifen | Breast cancer events, tumour characteristics, ER and PR status, CYP2D6 inhibitors | - Both PM and IM were not associated with early breast cancer events in a multivariable model compared to EM (adj. HR: 0.50; 95% CI: 0.07–3.82; *P*=0.50 for PM; adj. HR: 1.00; 95% CI: 0.47–2.11; *P*=1.00 for IM]. |
| Mayer et al., 2019 [44] | Seattle tri-county area | - Women diagnosed with invasive breast cancer with adjuvant TAM treatment (N=960) - Age at diagnosis: - Never use group:  45–54: 21.3%; 55–69: 44.9%; 70–79: 33.9%. - Use < 6 months group: 45–54: 19.3%, 55–69: 42.1%, 70–79: 38.6%. - Use 6+ months group: 45–54: 22.8%; 55–69: 42.9%; 70–79: 39.5% | - Cohort study - Timeframe for data collection: between 1993 and 1999 - Statistical analysis method: Cox models | BCSM | CYP2D6 | Tamoxifen | Age, BMI category, stage, grade, receipt of radiation and/or chemotherapy, and duration of adjuvant TAM | - No significant association between metabolizer phenotype and SBCE risk or BCSM - Use of CYP2D6 inhibitors was not associated with risk of SBCE or cancer mortality regardless of a women’s metabolic phenotype. |
| Moyer et al., 2011 [45] | The North Central Cancer Treatment Group | - Postmenopausal women with resected ER+ breast cancer treated with tamoxifen only with genotyping data available (N=190). - Age: <65 years 26.8%; ≥ 65 years 73.2% - Race: Caucasian 94.7%; African–American: 1.6%; American Indian or Alaska Native 0.5%; Not reported 3.2% | - Cohort study - Timeframe for data collection: January 1991 to April 1995 - Statistical analysis method: Log-rank test, the generalized Wilcoxon tests; Multivariate cox modeling | DFS | CYP2C19*17 | Tamoxifen | Tumour size and nodal status. | - DFS was not found to differ with respect to CYP2C19*17 genotype (*P*=0.667). This finding held after adjusting for tumour size and nodal status (HR: 0.93; 95% CI: 0.64–1.37). - In the subset of patients with impaired CYP2D6 activity, DFS was not found to differ with respect to CYP2C19*17 genotype (*P*=0.871, N=100). This finding held after adjusting for tumour size and nodal status (HR: 0.97; 95% CI: 0.59–1.59). |
| Newman et al., 2008 [46] | U.K. cancer genetics center, United Kingdom | - White Caucasian women with pathogenic BRCA1 or BRCA2 mutations with breast cancer and treated with tamoxifen (N=125) - Healthy ethnically matched control (N=90) | - Retrospective cohort study - Timeframe for data collection: Not mentioned - Statistical analysis method: Kaplan–Meier survival function with log-rank tests, Cox proportional hazard models | RFS, OS | CYP2D6*3, *4, *5 | Tamoxifen | Dose of  tamoxifen, duration of treatment, radiotherapy  and chemotherapy, tumour size and grade, ER status, and concomitant medication | - Patients with PM genotype had reduced OS (*P*=0.079; HR: 3.5; 95% CI: 0.8-15.4). - Patient group with reduced CYP2D6 activity due to concomitant use of an inhibitor had a trend to reduced overall survival (*P*=0.084; HR: 3.4; 95% CI: 0.77-14.9). |
| Nowell et al., 2005 [47] | Arkansas Cancer Research Center, University of Arkansas for Medical Sciences | - Primary invasive breast cancer patients who received tamoxifen (N=165) or not (N=172). - Age: <50 year: 139, >50 year: 198 - Race: Caucasian: 272 patients; African-American: 65 patients | - Cohort study - Timeframe for data collection: Between the years 1985 and 1996 - Statistical analysis method: Kaplan–Meier survival function with log-rank tests, Cox proportional hazard models | OS, PFS | CYP2D6*4 | Tamoxifen | Age, race, stage of disease at diagnosis, and hormone receptor status | - After adjusting for age, stage of disease, ethnicity and hormone receptor status, Cox proportional hazards modelling showed no association between CYP2D6*4 genotype and OS of breast cancer patients receiving tamoxifen (HR=0.77, 95% CI: 0.32–1.81). After excluding 11 patients who were never disease-free, there was no association between recurrence of disease and CYP2D6 genotype in tamoxifen-treated patients (HR=0.67, 95% CI: 0.33–1.35). |
| Okishiro et al., 2009 [48] | Not specified | - Primary breast cancer with ER- and/or PR- and treated with tamoxifen (N=173) - Tamoxifen alone (N=73); tamoxifen, chemotherapy, and/or goserelin (N=100) - Median age: 47, ranged 22-73. | - Cohort study - Timeframe for data collection: between October 1998 and December 2004 - Statistical analysis method: Kaplan-Meier method, Cox proportional hazard analyses | RFS | CYP2D6, CYP2C19 | Tamoxifen,  Goserelin | Tumour size, lymph node status, histologic grade, PR status, human epidermal growth factor receptor 2 status, and adjuvant therapy | - RFS rates were not significantly different between patients with the CYP2D6 *10/*10 genotype. - When the analysis was limited to patients who received adjuvant tamoxifen alone, there was no significant difference in RFS rates between these groups (log-rank test; *P*=0.57). - There still was no significant difference in RFS rates between patients with the CYP2D6 *10/*10 genotype after adjustment for other factors. |
| Park et al., 2011 [49] | Severance Hospital, Yonsei University Health System | - Patients with hormone receptor positive primary breast cancer who underwent surgery and adjuvant tamoxifen therapy (N=110) - Age: EM group: 42.8 ± 6.1; IM group: 44.2 ± 5.5; PM group: 40.8 ± 6.5 | - Cohort study - Timeframe for data collection: 2002 and 2004 - Statistical analysis method: Kaplan-Meier method and log rank test, Cox-proportional hazard model | RFS, OS | CYP2D6 | Tamoxifen | Age, tumour stage, nodal status, adjuvant chemotherapy status, and the status of extended or switching AI therapy | - The frequency of RFS and OS events among the EM, IM and PM groups were significantly different (RFS events; *P*=0.04, OS events; *P*=0.03). - No significant difference in RFS and OS between the EM and IM groups (*P*=0.96). - Poorer RFS and OS in PM group and it was statistically significant (EM vs PM in RFS; *P*=0.02, IM vs PM in RFS; *P*=0.01, EM vs PM in OS; *P*=0.01, IM vs PM in OS; *P*=0.008). - After adjusting for other factors, all metabolizer groups were not a statistically significant factor associated with RFS (HR=1.32, 95% CI: 0.25-6.88, *P*=0.74 for IM, HR=5.24, 95% CI: 0.70-39.13, *P*=0.10 for EM). |
| Park et al., 2012 [50] | National Cancer Center, Korea | - Primary breast cancer (stage I, II, or III) with ER+ and PR+ and received tamoxifen adjuvant therapy (N=716) - Age at diagnosis, years (median, range): 45 (24–78) - Duration of tamoxifen use: ＞6 months and ≤2 years: 106 (14.8%); ＞2 years: 610 (85.2%); Switch to AI: 212 (29.6%) | - Cohort study - Timeframe for data collection: January 2001 and December 2005. - Statistical analysis method: Kaplan–Meier method, Cox proportional hazards model | RFS | CYP2D6 | Tamoxifen, AI | Tumour stage, Ki-67, PR, HER2 status | - There was no CYP2D6 genotype-associated signiﬁcant difference in RFS. - Variant alleles of CYP2D6 were not related to RFS regardless of chemotherapy or switch to AI treatment. - Univariate analysis showed that V/V genotype was not statistically signiﬁcant for RFS on tamoxifen treatment in total patients with the Cox proportional hazard model (HR=1.14, 95% CI: 0.68-1.92, *P*=0.611). - Cox proportional hazard model estimates that the CYP2D6 variant type was not associated with RFS on tamoxifen treatment within any subgroup analysed. |
| Rae et al., 2012 [51] | ATAC trial | - Postmenopausal women in United Kingdom with invasive breast cancer (N=1203) with 10-year median follow-up | - Cohort study - Timeframe for data collection: July 1996 to March 2010 - Statistical analysis method: | DRFS | CYP2D6 | Tamoxifen | Tumour grade, tumour size, nadal status, hormone receptor status | - No statistically significant difference in the rates of distant recurrence was observed between CYP2D6 homozygous WT and metabolic variants. - After adjusting for concomitant medications, no statistically significant difference in rate of distant recurrence was observed between CYP2D6 phenotypes. |
| Ramón y Cajal et al., 2010 [52] | A single centre in Spain | - Primary invasive breast cancer patients with ER+ and received adjuvant monotherapy tamoxifen or tamoxifen with concomitant chemotherapy (N=91) - Age:28-79 - Ethnicity: Asian, Caucasian, African American | - Retrospective cohort study - Timeframe for data collection/analysis: 91 -133 months - Statistical analysis method of associative relationship: Kaplan Meier estimates, Log rank test, Cox Regression Model | DFS | CYP2D6 *3, *4, *5, *6, *20, *9, *10, *41, *1, *2, *35 | Tamoxifen, concomitant chemotherapy. | Cebrovascular accident, pulmonary  embolism, deep vein thrombosis, endometrial carcinoma,  toxicodermia, Intrauterine polyp, transaminitis, amenorrhea, steatosis and vaginitis | - No statistically significant difference in DFS between group A (IM/IM, EM/PM, EM/IM), Group B (PM/PM, PM/IM), and Group C (EM/EM, Ultra Extensive Metabolizers) (UM/EM), UM/IM) (P=0.413). - There was a statistically significant difference in DFS between group A (*4/*4, *4/*41, *1/*5 and *2/*5) and Group B (the remaining genotypes) (*P*=0.016). |
| Regan et al., 2012 [53] | BIG 1-98 Collaborative Group; International Breast Cancer Study Group (IBCSG); Danish Breast Cancer Collaborative Group | - Postmenopausal women with ER- or PgR-positive, operable invasive breast cancer and receive 5 years of monotherapy with tamoxifen or letrozole, or a sequential therapy of these drugs. (N=4861) - Age: median 61 (56-67) - Ethnicity: Not mentioned | - Cohort study - Timeframe for data collection/analysis: March 1998 and May 2008 - Statistical analysis method of associative relationship: Kaplan Meier estimates, Cox Regression Model | RFS | CYP2D6 | Tamoxifen and/or letrozole | Lymph node positivity, tumour size, tumour grade, ER and HER2 status | - Among tamoxifen-treated patients without previous chemotherapy, no association between CYP2D6 metabolism phenotype and Breast Cancer-Free Interval (BCFI) was noted (*P*=0.35) - Patients who were homozygous (CYP2D6*4/*4, analogous to the PM phenotype) or heterozygous (CYP2D6*4/WT, analogous to the IM phenotype) for CYP2D6*4 variant allele had risks of breast cancer events that were not statistically significantly different from patients who were homozygous for wild-type alleles (WT/WT, analogous to the EM phenotype) |
| Ruiter et al., 2010 [54] | Community in Holland | - ER+ breast cancer women on tamoxifen (N=80), not on tamoxifen (N=135) - Age: 55 years and above - Ethnicity: Caucasian origin | - Prospective cohort study - Timeframe for data collection/analysis: 3 to 4 years, or until recurrence, death, or end of the study - Statistical analysis method of associative relationship: Cox regression analysis, Kaplan Meier | BCS | CYP2C19 and CYP2D6 | Tamoxifen | None | - No association between genotype and breast cancer mortality in breast cancer patients not on tamoxifen (HR: 1.98, 95% CI: 0.56–7.43). - CYP2C19*2 carriers were associated with significantly longer BCS rate compared with wild type (HR 0.26, 95%CI: 0.08–0.87) in tamoxifen-treated group. |
| Saladores et al., 2015 [6] | Humphrey Oei Institute of Cancer  Research in Singapore;  Internal Medicine, American University of  Beirut in Lebanon; Cancer Sciences Academic Unit and University of Southampton Clinical  Trials Unit in University of Southampton, UK | - ER+ breast cancer premenopausal women (N=306) - Age: 22- 59 years - Ethnicity/race:   - Singaporean  - Lebanese  - Arabs, Caucasian of UK origin | - Prospective Cohort Study - Time frame for data collection/analysis: Not shown - Statistical analysis method of associative relationship: Cox Regression, Kaplan Meier, Log rank test | DRFS | CYP3A5, CYP2C9, CYP2C19, CYP2D6 | Tamoxifen | Non-genetic factors (age, BMI) | - Improved DRFS was associated with decreasing Tamoxifen metabolites (*P*=0.036) and increasing CYP2D6 activity score (HR=0.62, 95% CI: 0.43-0.91, *P*=0.014). - Low concentrations of endoxifen were associated with shorter DRFS (HR=1.94; 95% CI: 1.04–4.14; *P*=0.064). - PM patients had worse DRFS compared to EM subjects ([log-rank test, *P*=0.042]. |
| Sanchez-Spitman et al., 2019 [55] | Multicenter in the Netherlands and Belgium | - Early stage breast cancer women with ER+ status and treated with tamoxifen (N=667) - Age=18 and above - Ethnicity: Dutch and Belgian | - Prospective Clinical Trial - Timeframe for data collection/analysis: 0.10 to 9.30 years - Statistical analysis method of associative relationship: Kaplan-Meier Methods, Cox Regression, Exploratory Analysis | RFS, DFS, OS | CYP2D6 | Tamoxifen, aromatase Inhibitor | Endoxifen concentrations | - No significant differences in RFS between CYP2D6 phenotype groups (adj. HR: 0.929; 95% CI, 0.525-1.642, P = 0.799). - No association between RFS and CYP2D6 genotype from the start of tamoxifen therapy (UMs and EMs vs hetEM, IMs, and PMs (adj. HR: 0.962; 95% CI, 0.545-1.700; *P*=0.894) - No association between endoxifen concentrations and RFS [adj. HR: 0.991, 95% CI, 0.946 -1.038, *P*=0.691]. - No statistically significant association between endoxifen concentrations or CYP2D6 genotypes and the different end points (RFS, DFS, OS). |
| Schroth et al., 2007 [56] | Robert-Bosch Hospital Breast Center, Stuttgart, Germany | - Women with ER+ and primary invasive breast cancer (N=486): with tamoxifen (N=206); without tamoxifen (N=280) - Age: 29-92 - Ethnicity: Not mentioned | - Study design: Not mentioned - Data Collection and analysis: 4 to 227 months - Statistical analysis method: Cox Regression, Kaplan Meier, Logistic regression | RFS, OS | CYP2D6, CYP2C19, CYP3A5, CYP2B6, CYP2C9 | Tamoxifen | Tumour size, nodal status | - Significant shorter RFS in tamoxifen-treated patients with impaired functional CYP2D6 phenotype (HR=2.24; 95% CI: 1.16-4.33; *P*=0.02), and worse event-free survival rates (HR=1.89; 95% CI: 1.10-3.25; *P*=0.02). - Compared to patients with *1, *2, and *3 alleles, those with the CYP2C19 high enzyme activity promoter variant *17 had a more favourable clinical outcome (HR=0.45; 95% CI: 0.21-0.92; *P*=0.03). - Carriers of the *17 allele had longer OS rate compared to the rest, but was not a statistically significant difference (HR=0.61; 95% CI: 0.29 to 1.26; *P*=0.18). - No associations between genotypes and treatment outcome or survival were observed for the remaining polymorphisms of CYP2C19 (*2 and *3), CYP3A5, CYP2B6, and CYP2C9. |
| Schroth et al., 2009 [57] | German breast cancer cohort (Stuttgart, Karlsruhe, Erlangen, and Mainz) and the US North Central Cancer Treatment Group (NCCTG) (Mayo Clinic, Rochester, Minnesota) | - ER+ and PR+ early stage breast cancer women at stage I to III (N=1325) with no metastasis and no previous endocrine or chemotherapy at diagnosis - Age: 36.5- 93.1 years - Ethnicity/race: German, American | - Retrospective and prospective cohort - Time frame for data collection/analysis: 9 years - Statistical analysis method: Cox Regression model, Kaplan Meier, Log rank test | EFS, DFS, OS | CYP2D6 | Tamoxifen | None | - Those with decreased CYP2D6 activity (heterozygous EM/IM and PM) had worse EFS (HR=1.33; 95% CI: 1.06–1.68, *P*=0.01) and DFS (HR=1.29; 95% CI: 1.03–1.61, *P*=0.02) compared with EMs. - No significant difference in OS among groups (HR=1.13; 95% CI: 0.88–1.47, *P*=0.34). |
| Sensorn et al., 2013 [58] | Ramathibodi Hospital, Bangkok, Thailand | - Women with early stage breast cancer (N=30): ER+ (N=29) and ER- (N=1) - Age: 28- 74 years - Ethnicity/race: Thai | - Retrospective and prospective cohort study - Time frame for data collection/analysis: 3 years - Statistical analysis method of associative relationship: Cox Regression Analysis, Kaplan Meier, Log rank test | DFS | CYP3A4, CYP3A5 | Tamoxifen | None | - ABCB1 CT genotype patients had significantly shorter DFS than those with homozygous CC genotype (*P*=0.041). - Homozygous TT genotype patients showed no difference in DFS from wild-type CC patients (*P*=0.011). |
| Sensorn et al., 2016 [59] | Ramathibodi Hospital, Bangkok, Thailand | - Women with invasive breast cancer without metastasis and ER+ (N=73) - Age: 18 years and above - Ethnicity/race: Thai | - Retrospective Study - Time for data collection/analysis: 0.2 to 14.3 years - Statistical analysis method of association: Cox Regression Analysis, Kaplan Meier, Log rank test | DFS | CYP3A5, CYP2D6 | Tamoxifen | Tumour size, bone metastasis, lung and liver metastasis | - Patients carrying ABCC2 -24CC had a statistically significant shorter DFS than those who carry -24CT (log rank test, *P*=0.044. - The combination of genotypes of ABCC2 -24CC – ABCB1 3435 CT+TT was associated with increased risk of distant metastasis (Adj. HR=2.46; 95% CI: 1.15–5.26, *P*=0.020) |
| Sim et al., 2018 [60] | General practice  Hospital,  Sweden | - Breast cancer patients (N=306): - ER+: N=289, ER-: N=1, Unknown: N=16 - PR+: N=215, PR-: N=28, Unknown: N=63 - Age at diagnosis: 30-88 Year - Ethnicity/race: Swedish | - Cohort Study - Timeframe for data collection/analysis: not mentioned - Statistical analysis method of associative relationship: Cox regression Model, Kaplan Meier-log rank test | RFS, BCSS | CYP2C19, CYP2D6 | Tamoxifen | Menopausal status | - Effect of genotype predicted CYP2C19 and CYP2D6 phenotype combinations on RFS were statistically significant (*P*=0.025) and *P*=0.026 for BCSS. - Compared to premenopausal women with 2D6↓ versus 2D6↑, premenopausal women with 2C19↑–2D6↓ had a higher risk for poor RFS (HR=26.48, 95% CI: 4.55-154.28). - The 2C19↑–2D6↓ subgroup was associated with higher risk for poor RFS (HR=2.45,95% CI: 1.07-5.57) versus the remainder of patients. |
| Sirachainan et al., 2012 [61] | Ramathibodi Hospital, Thailand | - Breast cancer patients histologically diagnosed with ER+ and/or PR+, received tamoxifen as an adjuvant treatment and age at diagnosis >18 years old (N=39) - Case group: patients who had recurrence of breast cancer while receiving tamoxifen (N=20)   Control group: Patients who had already completed 5 years of adjuvant tamoxifen treatment (N=19) | - Case-control study - Timeframe for data collection: not mentioned - Statistical analysis method: Kaplan Meier, log rank test | DFS | CYP2D6*4 (1846G>A; rs3892097)  CYP2D6*5 (deletion)  CYP2D6*10 (100C>T, rs1065852) | Tamoxifen | None | - Patients with CYP2D6*10 homozygous variant (T/T; CYP2D6*10/*10) had a significantly shorter DFS than those with the heterozygous variant (C/T; CYP2D6 Wt/*10) (*P*=0.036). - There was a significant difference in DFS between homozygous wild-type (C/C; CYP2D6 Wt/Wt and heterozygous variant (C/T; CYP2D6 Wt/*10) (*P*=0.008). - No significant difference in DFS between heterozygous variants, homozygous variants (T/T; CYP2D6*10/*10), and homozygous wild-type patients (C/C; CYP2D6 Wt/Wt) (*P*=0.316). |
| Stingl et al., 2010 [62] | Division of Oncology, Department of Internal Medicine, Medical University, Graz | - Breast cancer patients without any other cancer diagnosis - Age: 59.2 (11.9) for CYP2D6*1/*1 group; 58.3 (11.9) for CYP2D6*1/*4 group; and 56.8 (10.1) for CYP2D6*4/*4 group - Race: Not mentioned | - Study design: retrospective analysis of a prospectively conducted trial - Timeframe for data collection: January 2020 to September 2004 - Timeframe for data analysis: Not mentioned. - Statistical analysis method of associative relationship: Wilcoxon test, Cox's proportional hazards regression analysis | PFS | CYP2D6*4 | Tamoxifen | Age at start of treatment, months of tamoxifen treatment, chemotherapy, tumour size, lymph nodes | - No significant difference in time to tumour progression or progression free survival between the CYP2D6*4 genotype groups in the overall study cohort was found. |
| Sukasem et al., 2012 [63] | Ramathibodi Hospital, Thailand | - Breast cancer patients (N=48): - ER+ (N=47), ER- (N=1); - PR+ (N=13), PR- (N=6); PR unknown (N=15) - Premenopause (N=30) - Post menopause (N=18) - Age: EM (28-72), IM (36-72) - Ethnicity/race: Thai | - Retrospective study - Timeframe for data collection/analysis: not mentioned - Statistical analysis method: Kaplan Meier, log rank test, Cox Proportional Regression, Exploratory analysis | DFS | CYP2D6 | Tamoxifen | None | - No association between CYDP2D6 and DFS. - Patients with IM phenotype had a statistically significant shorter DFS compared with AM phenotype in post menopause patients (HR=6.85; 95%CI: 1.48–31.69; *P=*0.005). - When compared to heterozygous CYP2D6*10 and other genotypes, homozygous CYP2D6*10 had statistically significant shorter DFS in post menopause patients (HR=10.52, 95%CI: 1.56-70.79, *P*=0.005). |
| Tamura et al., 2020 [64] | Recruited from 54 institutions in Japan | - Both premenopausal and postmenopausal women aged ≥ 20 years with ER+ breast cancer were enrolled (N=184) - 136 with wt/V or V/V were randomly assigned to receive regular dose of tamoxifen 20 mg daily (N=66), or receive increased dose of tamoxifen 40 mg daily (N=70) - Age: 61 (29-81) in regular dose tamoxifen group; 59 (31-90) in increased dose tamoxifen group - Ethnicity/race: Japanese | - Study design: a randomized, open-label phase II study. - Timeframe for data analysis/collection: December 2012 to July 2016. - Statistical analysis method: $\chi$^2^ tests, Wilcoxon tests, and Cox proportional hazards model | PFS | CYP2D6*2, *4, *6, *10, *14, *18, *21, *36, *41, *44 | Tamoxifen | None | - In patients with wt/V or V/V, the progression-free survival rates at 6 months did not differ significantly between patients receiving 20 mg tamoxifen daily (67.6%; 95% CI: 56.5% to 78.8%) and patients receiving 40 mg tamoxifen daily (66.7%; 95%CI: 55.0% to 78.3%). |
| Teh et al., 2012 [65] | Universiti Kebangsaan Malaysia Medical Centre,  Selayang Hospital and Tengku Ampuan Afzan Hospital | - ER+ breast cancer patients with tamoxifen (N=95) - Age: 33 to 79 years - Ethnicity/race: Malaysian (N=53), Chinese (N=36), Indians (N=6) | - Cohort study - Time for data collection/analysis: not mentioned - Statistical analysis method: Odds Ratios, Turkey’s Test, Kaplan Meier, Log rank test | RFS | CYP2D6*4, *xN, *5, *10,  *14 | Tamoxifen | None | - Carriers of CYP2D6*10/*10 and heterozygous null allele IM had higher risks of recurrence than CYP2D6*1/*1 and *1/*10 genotypes (OR=3.14, 95% CI: 1.57–109.94; *P*=0.004). - CYP2D6 IM and homozygous CC genotype of C3435T had statistically significant higher risks of recurrence (*P*=0.002). - Compared to CYP2D6 IM and homozygous CC genotype of C3435T combined, the median to time to recurrence was only 12 months (95% CI: 0.79–23.2) compared to those without this combination (48 months; 95% CI: 14.7–81.2). |
| Thompson et al., 2011 [66] | Two geographically  distinct cohorts in Dundee and Manchester, United Kingdom | - Postmenopausal and ER+ breast cancer patients treated with tamoxifen (N=618) - Age: Not specified - Ethnicity/race: British women/Caucasians | - Retrospective and prospective Cohort - Time for data collection/analysis: First 12 years of the study - Statistical analysis method: Cox Regression, Kaplan Meier, Log rank test | DFS | CYP2D6 | Tamoxifen | None | - Patients with one or more reduced functional or null alleles had reduced RFS (*P*=0.043). - Compared to patients with limited panel of reduced functional or null allele function of CYP2D6 to those classified as normal CYP2D6 function, no statistically significant results in RFS were found (*P*=0.39). |
| Toyama et al., 2009 [67] | Department of Breast and Endocrine Surgery, Nagoya  City University Hospital in Japan | - Node negative, breast cancer patients on tamoxifen (N=156): ER+ (N=148), ER- (N=8) - Age: 33 to 89 years - Ethnicity/race: Japanese women | - Study design: not mentioned - Time for data collection/analysis: 25 to 249 months - Statistical analysis method of associative relationship: X^2^, Kaplan Meier, Log rank test | DFS, DDFS, OS | CYP2D6*10 | Tamoxifen | None | - There were no correlations between CYP2D6 *10 genotypes and DFS, DDFS, OS. |
| Trojan et al., 2013 [68] | Hospital in Switzerland | - ER+ with stage I to III invasive breast cancer patients on tamoxifen (N=151) - Matched controls (N=148) - Age: 34 to 86 years - Ethnicity/race: Swiss women | - Matched Cohort Study - Time for data collection/analysis: 20 to 77 months - Statistical analysis method: Kaplan Meier, Log rank test | DFS | CYP2D6 | Tamoxifen | None | - Compared to controls, DFS of patients identified as EM CYP2D6 did not differ significantly (HR=0.42, 95%CI: 0.14-1.22; *P*=0.10). |
| Wegman et al., 2005 [69] | Community health facilities in South East Health Care  Region of Sweden | - ER+ with breast cancer patients on tamoxifen (N=677) - Age: 50 to 96 years - Ethnicity/race: Swedish women | - Randomized controlled Trial - Time for data collection/analysis: 0.4 to 17.9 years - Statistical analysis method of associative relationship: Kaplan Meier, Log rank test, Cox regression | RFS | CYP3A5, CYP2D6 | Tamoxifen | None | - For a group randomised to 5 years, tamoxifen survival showed a significantly improved RFS for patients with CYP3A5*3-homozygous patients (HR=0.20, 95% CI: 0.07 to 0.55, *P*=0.002). - There was a significant improved RFS with prolonged tamoxifen treatment in CYP3A5*3 homozygotes in multivariate Cox model (HR=0.13, 95% CI: 0.02-0.86, *P*=0.03), CYP2D6, SULT1A1 and UGT2B15 showed no differences in RFS. |
| Wegman et al., 2007 [70] | General practice, Sweden | - ER+ postmenopausal breast cancer patients with tamoxifen treatment (N=226) - Age: ≤70 years - Ethnicity/race: Swedish women | - Randomized controlled Trial - Time for data collection/analysis: 0.24 to 18.6 years - Statistical analysis method of associative relationship: Log rank Test, Cox regression, Relative risk Ratios | DRFS | CYP2D6*1, *4 | Tamoxifen adjuvant therapy  40 mg daily for 2 years | None | - Patients possessing at least one CYP2D6*4 allele had better DRFS when randomised to tamoxifen compared with those who were not randomised to tamoxifen (*P*=0.0089) |
| Xu et al., 2008 [71] | Breast Center at Peking University School of Oncology, China | - ER+ and ER- women with newly diagnosed breast cancer (N=293) - Age: 22-78 Years - Ethnicity/race: not mentioned | - Randomized controlled trial - Timeframe for data collection/analysis: Not specified - Statistical analysis method: Cox proportional regression, Kaplan Meier, Log rank test | DFS, DSS | CYP2D6 | Tamoxifen | None | - Women with homozygous variant T/T had a statistically significant lower 5-year DFS rate compared to homozygous wild type C/C or heterozygous C/T genotype, *P*=0.005). - Women with T/T genotype had a statistically significant worse DFS compared to C/C or C/T genotype in patients treated with tamoxifen (HR=2.7, 95%CI: 0.4–17.3, *P*=0.28). - T/T has significant effect on DFS inpatients treated with tamoxifen (HR=4.7; 95% CI: 1.1–20.0; *P*=0.04) - No significant association between CYP2D6*10 genotype and DFS as well as DSS in patients not treated with tamoxifen (*P*=0.99). - No statistically significant association between genotypes and DSF in women who were ER+ (*P*=0.18). |
| Zeng et al., 2017 [72] | Not mentioned | - ER+ breast cancer patients received treatment from Jan 2013 to Jan 2016 (N=78) - Age: average 58.4±7.9 | - Cohort study - Statistical analysis method: ANOVA, LSD-t method, X^2^ test | PFS | CYP2D6*1, *10 | Toremifene, tamoxifen | CA125, CA153, VEGF, IGF-1 | - Progression free survival in CYP2D6*10/*10 group was shortened with a higher recurrence rate and a lower survival rate. - There were no differences in comparison between CYP2D6*1/*1 group and CYP2D6*1/*10 group. |
| Zhang et al., 2015 [73] | The First Affiliated Hospital (Yijishan Hospital) of Wannan  Medical College, China | - ER+ and PR+ invasive breast cancer patients (N=296) - Age: 25 to 82 years - Ethnicity/race: Chinese Han women | - Cohort prospective study - Time for data collection/analysis: 9-155 months - Statistical analysis method: Kaplan Meier, Log rank Test, Cox regression | OS | CYP2D6*10 | Tamoxifen with Cyclophosphamide, Methotrexate or Fluorouracil | None | - No difference in overall survival between wild-type and carrier groups for CYP2D6*10 (P=0.096) - No statistical significance was observed between CYP2D6*10 wild-type and overall survival (HR=0.520, 95%CI: 0.237-1.139; *P*=0.102). |

Abbreviations: N: sample size, EM: Extensive metabolizers, IM: Intermediate metabolizers, PM: poor metabolizers, CYP: Cytochrome P450, PFS: Progression-free survival, OS: Overall survival, RFS: Recurrence-free survival, HR: Hazard Ratio, OR: Odds Ratio, CI: Confidence Interval, HER2: human epidermal growth factor receptor 2, ER: estrogen receptor, PR: progesterone receptor, GWAS: genome-wide association study, HR- positive: hormone receptor-positive, DDFS: Distant disease-free survival, DFS: disease-free survival, BCSM: breast cancer specific mortality, adj. HR: Multiple-adjusted hazard ratio, TOR: toremifene, TAM: tamoxifen, AIs: aromatase inhibitors, SBCEs: Second breast cancer events, IQR: interquartile range, TNM: Tumour Node metastasis, UM: Ultrarapid metabolizer, AS: activity score, RFT: Relapse-free time
